# Relaxin-2 plasma levels in atrial fibrillation are linked to inflammation and oxidative stress markers

**DOI:** 10.1038/s41598-022-26836-1

**Published:** 2022-12-24

**Authors:** Alana Aragón-Herrera, Marinela Couselo-Seijas, Sandra Feijóo-Bandín, Laura Anido-Varela, Sandra Moraña-Fernández, Estefanía Tarazón, Esther Roselló-Lletí, Manuel Portolés, José Luis Martínez-Sande, Javier García-Seara, Ezequiel Álvarez, José Ramón González-Juanatey, Moisés Rodríguez-Mañero, Sonia Eiras, Francisca Lago

**Affiliations:** 1 Cellular and Molecular Cardiology Unit and Department of Cardiology, Institute of Biomedical Research of Santiago de Compostela (IDIS-SERGAS), Travesía da Choupana s/n, 15706 Santiago de Compostela, Spain; 2 CIBERCV, Institute of Health Carlos III, C/ Monforte de Lemos 3-5, Pabellón 11, Planta 0, 28029 Madrid, Spain; 3 Translational Cardiology Group, Institute of Biomedical Research of Santiago de Compostela (IDIS-SERGAS), Travesía da Choupana s/n, 15706 Santiago de Compostela, Spain; 4 Cardiology Group, Center for Research in Molecular Medicine and Chronic Diseases (CIMUS), University of Santiago de Compostela and Health Research Institute, University Clinical Hospital of Santiago de Compostela, 15706 Santiago de Compostela, Spain; 5 Cardiocirculatory Unit, Health Institute La Fe University Hospital (IIS La Fe), Avda. de Fernando Abril Martorell 106, 46026 Valencia, Spain; 6 Arrhytmia Unit, University Clinical Hospital of Santiago de Compostela, Travesía da Choupana s/n, 15706 Santiago de Compostela, Spain; 7 Institute of Biomedical Research of Santiago de Compostela (IDIS-SERGAS), Travesía da Choupana s/n, 15706 Santiago de Compostela, Spain; 8 Department of Pharmacology, Pharmacy and Pharmaceutical Technology, University of Santiago de Compostela, 15782 Santiago de Compostela, Spain

**Keywords:** Biomarkers, Cardiology

## Abstract

Relaxin-2 exerts many favourable cardiovascular effects in pathological circumstances such as atrial fibrillation (AF) and heart failure, but the mechanisms underlying its actions are not completely understood. Since inflammation and fibrosis are pivotal processes in the pathogenesis of AF, our aim was to study the relationship between relaxin-2 plasma levels in left atrium (LA) and peripheral vein with molecules implicated in fibrosis, inflammation and oxidative stress in AF patients, and to evaluate the anti-fibrotic ability of relaxin-2 in normal human atrial cardiac fibroblasts (NHCF-A). Peripheral vein relaxin-2 plasma levels were higher than LA relaxin-2 plasma levels in men while, in women, peripheral vein relaxin-2 levels were increased compared to men. AF patients with higher levels of relaxin-2 exhibited a reduction in H_2_O_2_ plasma levels and in mRNA levels of *alpha-defensin 3* (*DEFA3*) and *IL-6* in leucocytes from LA plasma. Relaxin-2-in-vitro treatment inhibited NHCF-A migration and decreased mRNA and protein levels of the pro-fibrotic molecule transforming growth factor-β1 (TGF-β1). Our results support an association between relaxin-2 and molecules involved in fibrosis, inflammation and oxidative stress in AF patients, and reinforce an anti-fibrotic protective role of this hormone in NHCF-A; strengthening the relevance of relaxin-2 in AF physiopathology, diagnosis and treatment.

## Introduction

Nowadays, it is considered that the pleiotropic hormone relaxin-2 could be beneficial for atrial fibrillation (AF) management due to (a) its remarkable antiarrhythmic and cardioprotective properties, such as its anti-inflammatory, antiapoptotic, antifibrotic, anti-hypertrophic and antioxidant effects, (b) its capacity to inhibit angiotensin II (Ang II), or (c) its role regulating extracellular matrix (ECM) turn-over and reducing collagen excessive deposition in cardiac tissues^1–5^. Relaxin-2 seems to perform a relevant antifibrotic function through the regulation of cardiac fibroblast proliferation, the modulation of myofibroblasts differentiation and collagen synthesis, and also possibly through the control of the expression of profibrotic factors (including transforming growth factor-β1 (TGF-β1) or Ang II) and matrix metalloproteinases (MMPs)^1,2,6^. Some preclinical models have demonstrated that this hormone reduces AF susceptibility after myocardial infarction^3^, and in aged hypertensive rats^4,5^. One of the mechanisms suggested to be involved in this effect might be the regulation of ionic currents and inotropy in cardiac cells^4,7^.

Patients with persistent AF or recurrence after radiofrequency catheter ablation show an increase in plasma relaxin-2 levels, that, in some cases, positively correlate with tumor necrosis factor-α (TNF-α), TGF-β, procollagen type I C-terminal peptide (PICP), and left atrium (LA) diameter^8,9^. The involvement of these molecules in fibrosis suggests a tight relationship between relaxin-2, fibrosis and AF^8,9^. The fact that the recombinant form of relaxin-2, known as serelaxin, was shown to be safe in acute heart failure (AHF) patients in the phase III clinical trial RELAXin in Acute Heart Failure (RELAX-AHF) constitutes an additional advantage and encourages the possible use of this hormone as a therapeutic strategy for AF^10,11^, since 53% of the participants had a previous clinical history with AF, 41% presented AF during the course of the clinical trial, and serelaxin treatment was associated with a slight decrease in AF incidence^10–12^. Unfortunately, at present, there are no specific clinical trials studying the effect of the treatment with this hormone in the context of AF. The outstanding biological role of relaxin-2, along with the numerous basic, clinic, and translational findings showing its beneficial cardiovascular and electrophysiological properties, sustain that it is possible to consider that this hormone could be useful as a biomarker in the diagnosis, prediction, risk stratification and management of AF^5,8,9,13^. In this study, our purpose was to characterize the endogenous plasmatic levels of relaxin-2 in patients with AF, as well as its possible association with molecules implicated in fibrotic, inflammatory and oxidative stress mechanisms, all of them closely related to the physiopathology of AF^14^.

## Results

### LA and peripheral vein plasmatic levels of relaxin-2 in patients with AF

**Figure 1:**
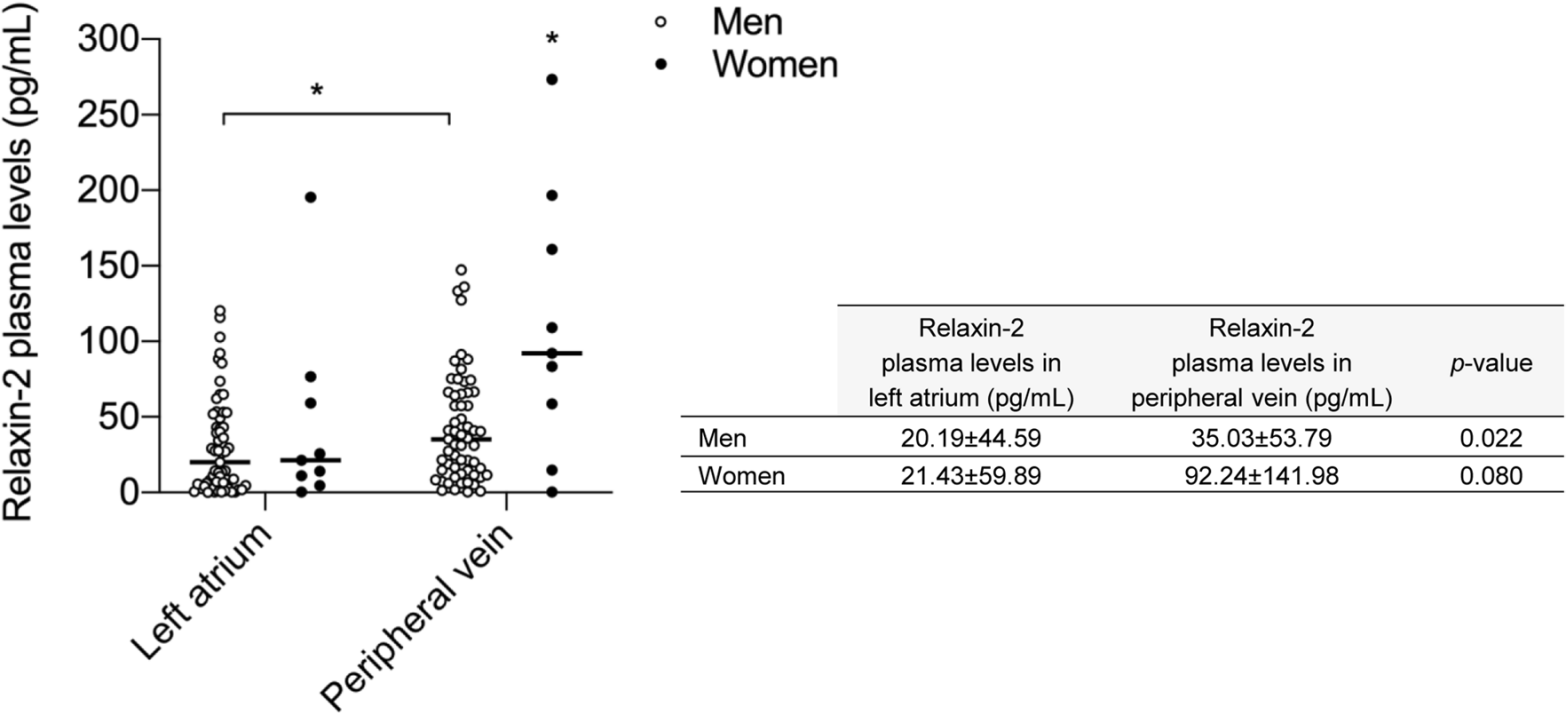
Dispersion graph showing the individual values of relaxin-2 plasma levels in left atrium and peripheral vein of patients with atrial fibrillation (AF) depending on the gender and panel with plasma relaxin-2 concentrations determined. The bracket with the symbol ‘*’ denotes that relaxin-2 plasma levels in peripheral vein are significantly higher than relaxin-2 plasma levels in left atrium in men. The symbol ‘*’ indicates that relaxin-2 plasma levels in peripheral vein of women are significantly elevated than relaxin-2 plasma levels in peripheral vein of men. Horizontal bar represents the median value of relaxin-2 plasma levels in left atrium and peripheral vein of men and women. Plasma relaxin-2 concentrations in left atrium and peripheral vein of patients with AF depending on the gender in the right panel are expressed as median ± interquartile range (IQR). n_men_ = 59, n_women_ = 9. Statistical analysis: Mann–Whitney U test. *p-value < 0.05.

We determined LA and peripheral vein plasma levels of relaxin-2 in 68 consecutive patients (59 men and 9 women) with AF. Men reached a relaxin-2 concentration (expressed in median ± IQR) of 20.19 ± 44.59 pg/mL and 35.03 ± 53.79 pg/mL in LA and peripheral vein, respectively. Women showed a relaxin-2 concentration of 21.43 ± 59.89 pg/mL in LA and of 92.24 ± 141.98 pg/mL in peripheral vein. The whole population showed substantial differences in peripheral vein relaxin-2 plasma levels regarding gender. Similar levels between men and women were found referring to LA relaxin-2 levels. However, the peripheral vein relaxin-2 levels in women were higher than in men (*p*-value = 0.014) (Fig. 1). Among men, relaxin-2 plasma levels in peripheral vein were significantly increased compared to relaxin-2 plasma levels in LA (*p*-value = 0.022) (Fig. 1). In our study, from a total of 68 patients, 4 patients (5.90% of the patients) had indetectable levels of relaxin-2 in LA, and only 2 patients (2.90% of the patients) had indetectable levels of relaxin-2 in peripheral vein.

### Patient’s general features according to relaxin-2 subgroups

A cut-off value of relaxin-2, defined by the median value of relaxin-2 plasma levels in LA and peripheral vein, was used for grouping the patients with high or low LA and peripheral vein plasma relaxin-2 levels. We did not find statistical differences regarding gender, age, BMI, AHT, type 2 diabetes mellitus (T2DM) or chronic kidney disease (CKD). Neither the glucose, total cholesterol (TC), low-density lipoprotein cholesterol (LDL-c), high-density lipoprotein cholesterol (HDL-c), triglycerides (TG), left ventricular ejection fraction (LVEF), heart rate (HR), LA area, or type of AF were statistically different between subgroups. The number of smokers was significantly higher in the subgroup of patients with LA relaxin-2 plasma levels above the median value (*p*-value = 0.043). Patients’ general characteristics at admission between subgroups are shown in Table 1. In addition, no statistical differences were observed when the initial population was separated according to gender (Tables 2 and 3).

**Table 1:** General characteristics of patients with atrial fibrillation according to relaxin-2 subgroups.

	All patients (n = 68)	Left atrium relaxin-2 plasma levels			Peripheral vein relaxin-2 plasma levels		
		≤ 20.81 ± 46.21 pg/mL (n = 34)	> 20.81 ± 46.21 pg/mL (n = 34)	*p*-value	≤ 39.32 ± 59.47 pg/mL (n = 34)	> 39.32 ± 59.47 pg/mL (n = 34)	*p*-value
Male, n (%)	59 (86.8%)	30 (88.2%)	29 (85.3%)	1.000	32 (94.1%)	27 (79.4%)	0.150
Female, n (%)	9 (13.2%)	4 (11.8%)	5 (14.7%)	1.000	2 (5.9%)	7 (20.6%)	0.150
Age (years), median ± IQR	56.50 ± 14.50	56.50 ± 13.25	57.00 ± 14.50	0.980	56.00 ± 13.75	58.00 ± 15.75	0.547
BMI (kg/m^2^), median ± IQR	29.38 ± 6.08	28.73 ± 6.66	29.44 ± 5.94	0.229	28.73 ± 6.66	29.44 ± 6.65	0.333
AHT, n (%)	25 (36.80%)	13 (38.20%)	12 (35.30%)	1.000	12 (35.30%)	13 (38.20%)	1.000
T2DM, n (%)	6 (8.80%)	2 (5.90%)	4 (11.80%)	0.673	2 (5.90%)	4 (11.80%)	0.673
CKD, n (%)	2 (2.90%)	1 (2.90%)	1 (2.90%)	1.000	1 (2.90%)	1 (2.90%)	1.000
Obesity, n (%)	30 (44.10%) (n = 67)	15 (44.10%) (n = 33)	15 (44.10%)	1.000	14 (41.20%)	16 (47.10%) (n = 33)	0.627
Smokers, n (%)	16 (23.50%)	**4 (11.80%)**	**12 (35.30%)**	**0.043**	5 (14.70%)	11 (32.40%)	0.152
Glucose (mg/dL), median ± IQR	100.50 ± 21.50	98.50 ± 20.00	103.00 ± 23.25	0.628	99.50 ± 20.00	102.00 ± 24.25	0.602
TC (mg/dL), median ± IQR	193.00 ± 59.50 (n = 65)	193.50 ± 62.50 (n = 32)	193.00 ± 50.00 (n = 33)	0.443	195.00 ± 78.00 (n = 32)	193.00 ± 50.00 (n = 33)	0.341
LDL-c (mg/dL), median ± IQR	120.50 ± 48.75 (n = 64)	121.00 ± 47.00 (n = 31)	115.00 ± 49.50 (n = 33)	0.600	121.00 ± 49.00 (n = 31)	120.00 ± 48.00 (n = 33)	0.633
HDL-c (mg/dL), median ± IQR	47.50 ± 21.50 (n = 64)	49.00 ± 22.00 (n = 31)	45.00 ± 19.00 (n = 33)	0.489	50.00 ± 23.00 (n = 31)	45.00 ± 17.00 (n = 33)	0.256
TG (mg/dL), median ± IQR	116.00 ± 77.50 (n = 65)	115.50 ± 71.00 (n = 32)	123.00 ± 83.00 (n = 33)	0.808	112.50 ± 86.25 (n = 32)	123.00 ± 67.00 (n = 33)	0.778
LVEF (%), median ± IQR	64.00 ± 11.00	63.00 ± 10.00	66.00 ± 10.75	0.210	63.00 ± 12.25	64.50 ± 9.50	0.663
HR (bpm), median ± IQR	67.50 ± 21.50	68.00 ± 21.15	65.50 ± 25.25	0.740	71.00 ± 25.25	65.50 ± 18.00	0.572
LA Area (cm^2^), median ± IQR	8.11 ± 2.59	8.06 ± 2.07	8.33 ± 7.87	0.528	8.06 ± 2.19	8.30 ± 2.97	0.481
LA Volume (mL), median ± IQR	35.84 ± 24.87 (n = 66)	34.67 ± 23.09 (n = 33)	39.27 ± 26.28 (n = 33)	0.492	32.18 ± 22.93 (n = 32)	40.79 ± 24.25	1.000
**Type of AF, n (%)**							
Paroxysmal	23 (33.80%)	12 (35.30%)	11 (32.40%)	1.000	12 (35.30%)	11 (32.40%)	1.000
Persistent	29 (42.60%)	14 (41.20%)	15 (44.10%)	1.000	13 (38.20%)	16 (47.10%)	0.624
Long-standing persistent	16 (23.50%)	8 (23.50%)	8 (23.50%)	1.000	9 (26.50%)	7 (20.60%)	0.776
AF duration (months), median ± IQR	36.00 ± 51.00 (n = 67)	36.00 ± 50.00 (n = 33)	36.00 ± 54.00 (n = 34)	0.905	36.00 ± 59.00 (n = 33)	36.00 ± 47.00 (n = 34)	0.702

The exact *n* values are stated in the table depending on the subgroups and the different parameters. Values are represented as *n* (%) or median ± Interquartile Range (IQR) in %, pg/mL, years, kg/m^2^, mg/dL, bpm, cm^2^, mL or months. Statistical analysis: Fisher’s exact test and Mann–Whitney U test. *p*-value for the difference between patient groups defined according to the median value of relaxin-2 distribution in left atrium and peripheral vein.

AHT: arterial hypertension; BMI: body mass index; bpm: beats per minute; CKD: chronic kidney disease; HDL-c: high-density lipoprotein cholesterol; HR: heart rate; IQR: interquartile range; LA: left atrium; LDL-c: low-density lipoprotein cholesterol; LVEF: left ventricular ejection fraction; T2DM: type 2 diabetes mellitus; TC: total cholesterol; TG: triglycerides.

Significant values are in bold.

**Table 2:** General characteristics of male patients with atrial fibrillation according to relaxin-2 subgroups.

	Men						
	All patients (n = 59)	Left atrium relaxin-2 plasma levels			Peripheral vein relaxin-2 plasma levels		
		≤ 20.19 ± 44.59 pg/mL (n = 29)	> 20.19 ± 44.59 pg/mL (n = 30)	*p*-value	≤ 35.03 ± 53.79 pg/mL (n = 29)	> 35.03 ± 53.79 pg/mL (n = 30)	*p*-value
Age (years), median ± IQR	56.00 ± 14.00	56.00 ± 12.50	57.00 ± 14.00	0.808	56.00 ± 12.00	57.00 ± 14.00	0.756
BMI (kg/m^2^), median ± IQR	29.37 ± 6.18	29.07 ± 6.03	29.38 ± 6.59	0.868	29.05 ± 5.44	29.40 ± 7.48	0.671
AHT, n (%)	20 (33.90%)	11 (37.90%)	9 (30.00%)	0.589	10 (34.50%)	10 (34.50%)	1.000
T2DM, n (%)	6 (10.2%)	2 (6.90%)	4 (13.30%)	0.671	1 (3.40%)	5 (16.70%)	0.195
CKD, n (%)	1 (1.70%)	0 (0.00%)	1 (3.30%)	1.000	0 (0.00%)	1 (3.30%)	1.000
Obesity, n (%)	25 (42.40%) (n = 58)	13 (44.80%) (n = 28)	12 (40.00%)	0.791	12 (41.40%)	13 (43.30%) (n = 29)	1.000
Smokers, n (%)	15 (25.40%)	4 (13.80%)	11 (36.70%)	0.072	4 (13.80%)	11 (36.70%)	0.072
Glucose (mg/dL), median ± IQR	101.00 ± 20.00	98.00 ± 19.00	104.50 ± 20.25	0.098	99.00 ± 20.50	103.00 ± 22.50	0.976
TC (mg/dL), median ± IQR	190.00 ± 61.75 (n = 56)	190.00 ± 59.00 (n = 27)	186.00 ± 55.50 (n = 29)	0.634	190.00 ± 66.00 (n = 27)	189.00 ± 61.50 (n = 29)	0.394
LDL-c (mg/dL), median ± IQR	117.00 ± 50.00 (n = 55)	118.50 ± 49.75 (n = 26)	115.00 ± 53.00 (n = 29)	0.730	117.00 ± 53.25 (n = 26)	115.00 ± 52.00 (n = 29)	0.527
HDL-c (mg/dL), median ± IQR	45.00 ± 15.00 (n = 55)	46.50 ± 15.75 (n = 26)	43.00 ± 14.50 (n = 29)	0.649	49.50 ± 18.25 (n = 26)	43.00 ± 11.00 (n = 29)	0.084
TG (mg/dL), median ± IQR	123.00 ± 82.25 (n = 56)	116.00 ± 59.00 (n = 27)	123.00 ± 95.50 (n = 29)	0.664	104.00 ± 79.00 (n = 27)	136.00 ± 74.50 (n = 29)	0.321
LVEF (%), median ± IQR	64.00 ± 12.00	63.00 ± 11.00	66.00 ± 13.00	0.400	63.00 ± 12.50	65.00 ± 10.75	0.404
HR (bpm), median ± IQR	68.00 ± 25.00	74.00 ± 25.00	66.00 ± 25.75	0.638	74.00 ± 26.00	67.50 ± 20.50	0.832
LA Area (cm^2^), median ± IQR	8.14 ± 2.45	8.07 ± 2.09	8.33 ± 3.19	0.500	8.00 ± 2.45	8.53 ± 2.89	0.163
LA Volume (mL), median ± IQ R	33.33 ± 23.41 (n = 57)	32.18 ± 21.57 (n = 28)	38.38 ± 24.19 (n = 29)	1.000	32.08 ± 19.05 (n = 27)	36.97 ± 23.28	1.000
**Type of AF, n (%)**							
Paroxysmal	18 (30.50%)	8 (27.60%)	10 (33.30%)	0.779	8 (27.60%)	10 (33.30%)	0.779
Persistent	27 (45.80%)	14 (48.30%)	13 (43.30%)	0.796	13 (44.80%)	14 (46.70%)	1.000
Long-standing persistent	14 (23.70%)	7 (24.10%)	7 (23.30%)	1.000	8 (27.60%)	6 (20.00%)	0.552
AF duration (months), median ± IQR	36.00 ± 52.00 (n = 58)	32.00 ± 60.00 (n = 28)	36.00 ± 53.00	0.975	25.50 ± 60.00 (n = 28)	36.00 ± 52.00	0.919

The exact *n* values are stated in the table depending on the subgroups and the different parameters. Values are represented as *n* (%) or median ± Interquartile Range (IQR) in %, pg/mL, years, kg/m^2^, mg/dL, bpm, cm^2^, mL or months. Statistical analysis: Fisher’s exact test and Mann–Whitney U test. *p*-value for the difference between patient groups defined according to the median value of relaxin-2 distribution in left atrium and peripheral vein.

AHT: arterial hypertension; BMI: body mass index; bpm: beats per minute; CKD: chronic kidney disease; HDL-c: high-density lipoprotein cholesterol; HR: heart rate; IQR: interquartile range; LA: left atrium; LDL-c: low-density lipoprotein cholesterol; LVEF: left ventricular ejection fraction; T2DM: type 2 diabetes mellitus; TC: total cholesterol; TG: triglycerides.

**Table 3:** General characteristics of female patients with atrial fibrillation according to relaxin-2 subgroups.

	Women						
	All patients (n = 9)	Left atrium relaxin-2 plasma levels			Peripheral vein relaxin-2 plasma levels		
		≤ 21.43 ± 59.89 pg/mL (n = 5)	> 21.43 ± 59.89 pg/mL (n = 4)	*p*-value	≤ 92.24 ± 141.98 pg/mL (n = 4)	> 92.24 ± 141.98 pg/mL (n = 5)	*p*-value
Age (years), median ± IQR	66.00 ± 12.00	66.00 ± 12.00	60.50 ± 29.75	0.706	60.50 ± 15.50	66.00 ± 21.50	0.900
BMI (kg/m^2^), median ± IQR	29.48 ± 7.90	26.23 ± 8.47	31.67 ± 15.39	0.086	26.61 ± 10.94	29.48 ± 10.13	0.462
AHT, n (%)	5 (55.60%)	3 (60.00%)	2 (50.00%)	1.000	2 (50.00%)	3 (60.00%)	1.000
T2DM, n (%)	0	0	0	-	0	0	-
CKD, n (%)	1 (11.10%)	1 (20.00%)	0 (0.00%)	1.000	1 (25.00%)	0 (0.00%)	0.444
Obesity, n (%)	5 (55.60%)	2 (40.00%)	3 (75.00%)	0.524	2 (50.00%)	3 (60.00%)	1.000
Smokers, n (%)	1 (11.1%)	0 (0.00%)	1 (25.00%)	0.444	0 (0.00%)	1 (20.00%)	1.000
Glucose (mg/dL), median ± IQR	100.00 ± 23.50	101.00 ± 20.50	89.00 ± 39.50	0.623	105.00 ± 20.00	88.00 ± 35.00	0.219
TC (mg/dL), median ± IQR	216.00 ± 73.50	256.00 ± 73.50	194.00 ± 50.25	0.086	231.00 ± 67.25	216.00 ± 83.50	0.624
LDL-c (mg/dL), median ± IQR	126.00 ± 29.00	126.00 ± 36.50	111.50 ± 43.00	0.462	126.50 ± 11.50	122.00 ± 65.50	0.806
HDL-c (mg/dL), median ± IQR	67.00 ± 18.00	78.00 ± 28.50	66.50 ± 13.75	0.085	76.50 ± 38.50	67.00 ± 18.50	0.389
TG (mg/dL), median ± IQR	112.00 ± 68.00	115.00 ± 115.50	103.50 ± 60.50	0.327	88.50 ± 143.00	112.00 ± 50.00	0.624
LVEF (%), median ± IQR	64.00 ± 4.50	64.00 ± 3.00	64.50 ± 15.50	0.902	63.50 ± 4.00	65.00 ± 11.50	0.902
HR (bpm), median ± IQR	63.00 ± 25.00	63.00 ± 31.50	61.00 ± 23.50	0.624	62.00 ± 33.00	63.00 ± 24.50	1.000
LA Area (cm^2^), median ± IQR	7.74 ± 4.74	8.75 ± 6.26	7.26 ± 2.57	0.327	9.24 ± 4.56	6.83 ± 3.93	0.086
LA Volume (mL), median ± IQR	44.05 ± 47.88	50.00 ± 41.51	36.60 ± 45.79	1.000	61.52 ± 31.16	30.77 ± 37.85	1.000
**Type of AF, n (%)**							
Paroxysmal	5 (55.60%)	3 (60.00%)	2 (50.00%)	1.000	3 (75.00%)	2 (40.00%)	0.524
Persistent	2 (22.20%)	1 (20.00%)	1 (25.00%)	1.000	0 (0.00%)	2 (40.00%)	0.444
Long-standing persistent	2 (22.20%)	1 (20.00%)	1 (25.00%)	1.000	1 (25.00%)	1 (20.00%)	1.000
AF Duration (months), median ± IQR	36.00 ± 45.00	36.00 ± 45.00	30.00 ± 155.00	0.902	42.00 ± 44.00	18.00 ± 114.00	0.712

The exact *n* values are stated in the table depending on the subgroups and the different parameters. Values are represented as *n* (%) or median ± Interquartile Range (IQR) in %, pg/mL, years, kg/m^2^, mg/dL, bpm, cm^2^, mL or months. Statistical analysis: Fisher’s exact test and Mann–Whitney U test. *p*-value for the difference between patient groups defined according to the median value of relaxin-2 distribution in left atrium and peripheral vein.

AHT: arterial hypertension; BMI: body mass index; bpm: beats per minute; CKD: chronic kidney disease; HDL-c: high-density lipoprotein cholesterol; HR: heart rate; IQR: interquartile range; LA: left atrium; LDL-c: low-density lipoprotein cholesterol; LVEF: left ventricular ejection fraction; T2DM: type 2 diabetes mellitus; TC: total cholesterol; TG: triglycerides.

### Association between relaxin-2 plasma levels with plasmatic levels of molecules implicated in fibrotic, inflammatory, and oxidative stress mechanisms in patients with AF

Patients with relaxin-2 concentrations in peripheral vein above the median had significantly higher levels of Gal-3 in peripheral vein plasma (*p*-value = 0.022) than patients with relaxin-2 concentrations below the median (Table 4). On the contrary, *DEFA3* and *IL-6* mRNA expression levels in leucocytes from LA plasma were significantly lower in patients with higher relaxin-2 levels in LA (*p*-value = 0.034 for *DEFA3*, and *p*-value = 0.003 for *IL-6*) and in peripheral vein (*p*-value = 0.001 for *DEFA3*, and *p*-value = 0.001 for *IL-6*) (Table 4). Moreover, peripheral vein plasma levels of H_2_O_2_ tended to decrease (*p*-value = 0.065) in patients with high relaxin-2 plasma levels in peripheral vein (Table 4).

**Table 4:** Fibrotic, inflammatory and oxidative stress molecules, and markers of left-atrial voltage mapping in patients with atrial fibrillation (AF) according to relaxin-2 subgroups.

	All patients (n = 68)	Left atrium relaxin-2 plasma levels			Peripheral vein relaxin-2 plasma levels		
		≤ 20.81 ± 46.21 pg/mL (n = 34)	> 20.81 ± 46.21 pg/mL (n = 34)	*p*-value	≤ 39.32 ± 59.47 pg/mL (n = 34)	> 39.32 ± 59.47 pg/mL (n = 34)	*p*-value
LA Gal-3 (ng/mL), median ± IQR	13.21 ± 6.09 (n = 64)	11.57 ± 6.56 (n = 31)	14.21 ± 6.59 (n = 33)	0.069	11.57 ± 6.47 (n = 31)	14.27 ± 5.72 (n = 33)	0.073
Peripheral vein Gal-3 (ng/mL), median ± IQR	14.21 ± 5.18 (n = 64)	13.50 ± 6.86 (n = 31)	14.84 ± 4.28 (n = 33)	0.056	**13.27 ± 6.39 (n = 31)**	**14.84 ± 4.09 (n = 33)**	**0.022**
LA DEFA3 (a.u), median ± IQR	1.82 ± 0.17	**1.84 ± 0.17**	**1.80 ± 0.16**	**0.034**	**1.85 ± 0.15**	**1.77 ± 0.17**	**0.001**
LA IL-6 (a.u), median ± IQR	1.45 ± 0.08 (n = 67)	**1.48 ± 0.08 (n = 33)**	**1.43 ± 0.05**	**0.003**	**1.48 ± 0.05 (n = 33)**	**1.42 ± 0.05**	**0.001**
LA H_2_O_2_ (μM), median ± IQR	0.12 ± 0.23 (n = 64)	0.13 ± 0.27	0.12 ± 0.17 (n = 30)	0.370	0.13 ± 0.27	0.11 ± 0.17 (n = 30)	0.126
Peripheral vein H_2_O_2_ (μM), median ± IQR	0.11 ± 0.09 (n = 64)	0.12 ± 0.07	0.09 ± 0.11 (n = 30)	0.562	0.12 ± 0.08	0.09 ± 0.08 (n = 30)	0.065
**LA voltage mapping (cm**^**2**^**), median ± IQR**							
Total area of scar	213.90 ± 63.90 (n = 64)	201.10 ± 56.63 (n = 30)	223.85 ± 68.53	1.000	212.85 ± 66.15 (n = 30)	220.65 ± 65.07	1.000
Atrial area of scar	94.55 ± 37.13 (n = 64)	84.00 ± 37.15 (n = 30)	99.40 ± 37.87	0.870	94.55 ± 33.30 (n = 30)	95.15 ± 41.52	0.491
Health area (> 1.5 mV)	76.80 ± 36.30 (n = 64)	67.70 ± 45.85 (n = 30)	85.80 ± 33.18	1.000	76.80 ± 41.50 (n = 30)	75.70 ± 36.37	0.744
TrZ area (0.5–1.5 mV)	7.75 ± 13.48 (n = 64)	**6.50 ± 13.50 (n = 30)**	**7.85 ± 13.88**	**0.041**	7.45 ± 14.18 (n = 30)	7.85 ± 13.28	0.644
LVZ area (< 0.5 mV)	1.65 ± 7.65 (n = 64)	2.20 ± 12.18 (n = 30)	0.95 ± 5.00	0.659	2.20 ± 9.15 (n = 30)	1.40 ± 6.75	0.806

Galectin-3 (Gal-3) and H_2_O_2_ plasma levels in left atrium (LA) and peripheral vein in all patients with AF and in relaxin-2 LA and peripheral vein subgroups according to the median relaxin-2 distribution. *Alpha defensin 3* (*DEFA3*) and *interleukin-6* (*IL-6*) expression levels in leucocytes from LA plasma in all patients with AF and in relaxin-2 LA and peripheral vein subgroups according to the median value of relaxin-2. Values are represented as median ± Interquartile Range (IQR) in pg/mL, ng/mL, arbitrary units (a.u.), μM or cm^2^. The exact *n* values are stated in the table depending on the subgroups and the molecule studied. Statistical analysis: Mann–Whitney U test. *p*-value for the difference between patient groups defined according to the median value of relaxin-2 distribution in LA and peripheral vein.

AF: atrial fibrillation; DEFA3: alpha defensin 3; Gal-3: galectin-3; IL-6: interleukin-6; IQR: interquartile range; LA: left atrium; LVZ: low-voltage zones; PV: pulmonary vein; TrZ: transition zone.

Significant values are in bold.

*p-value < 0.05, **p-value < 0.01, ***p-value < 0.001.

Among men (n = 59), Gal-3 plasma levels in LA (*p*-value = 0.041) and peripheral vein (*p*-value = 0.009) were significantly higher in AF patients with peripheral vein relaxin-2 plasma levels above the median (Table 5). However, the association between Gal-3 and relaxin-2 was lost after including the confounding factors (age, BMI and AHT) in a logistic regression analysis (Supplementary Tables S1 and Table S2 online). Male patients with LA and peripheral vein relaxin-2 plasma levels above the median showed a significant decrease in *DEFA3* (*p*-value = 0.019 in LA, and *p*-value = 0.004 in peripheral vein) and *IL-6* (*p*-value = 0.002 in LA, and *p*-value = 0.000 in peripheral vein) mRNA expression levels in leucocytes from LA plasma (Table 5). In addition, men with high peripheral vein relaxin-2 plasma levels, showed a significant decrease (*p*-value = 0.002) in peripheral vein H_2_O_2_ plasma levels (Table 5). The logistic regression analysis has shown a significant association between these biomarkers and relaxin-2 (while also considering age, BMI and AHT as covariates) (Supplementary Tables S3, S4, S5, S6 and S7 online).

**Table 5:** Fibrotic, inflammatory and oxidative stress molecules, and markers of left-atrial voltage mapping in men patients with atrial fibrillation (AF) according to relaxin-2 subgroups.

	Men						
	All patients (n = 59)	Left atrium relaxin-2 plasma levels			Peripheral vein relaxin-2 plasma levels		
		≤ 20.19 ± 44.59 pg/mL (n = 29)	> 20.19 ± 44.59 pg/mL (n = 30)	*p*-value	≤ 35.03 ± 53.79 pg/mL (n = 29)	> 35.03 ± 53.79 pg/mL (n = 30)	*p*-value
LA Gal-3 (ng/mL), median ± IQR	12.72 ± 5.80 (n = 55)	10.59 ± 6.20 (n = 27)	13.63 ± 6.00 (n = 28)	0.074	**10.60 ± 5.98 (n = 26)**	**13.83 ± 5.94 (n = 29)**	**0.041**
Peripheral vein Gal-3 (ng/mL), median ± IQR	14.18 ± 5.29 (n = 55)	13.50 ± 6.59 (n = 27)	15.02 ± 4.64 (n = 28)	0.072	**12.93 ± 4.79 (n = 26)**	**15.61 ± 5.27 (n = 29)**	**0.009**
LA DEFA3 (a.u), median ± IQR	1.84 ± 0.15	**1.86 ± 0.13**	**1.82 ± 0.11**	**0.019**	**1.86 ± 0.13**	**1.82 ± 0.13**	**0.004**
LA IL-6 (a.u), median ± IQR	1.46 ± 0.08 (n = 58)	**1.48 ± 0.06 (n = 28)**	**1.43 ± 0.05**	**0.002**	**1.48 ± 0.06 (n = 28)**	**1.43 ± 0.06**	**0.000**
LA H_2_O_2_ (μM), median ± IQR	0.12 ± 0.19 (n = 55)	0.13 ± 0.23	0.12 ± 0.18 (n = 26)	0.327	0.13 ± 0.29	0.11 ± 0.12 (n = 26)	0.073
Peripheral vein H_2_O_2_ (μM), median ± IQR	0.11 ± 0.10 (n = 55)	0.12 ± 0.07	0.09 ± 0.13 (n = 26)	0.211	**0.13 ± 0.09**	**0.07 ± 0.08 (n = 26)**	**0.002**
**LA voltage mapping (cm**^**2**^**), median ± IQR**							
Total area of scar	219.20 ± 61.60 (n = 55)	215.05 ± 55.35 (n = 26)	224.30 ± 64.85 (n = 29)	1.000	213.60 ± 62.70 (n = 25)	223.85 ± 60.67	1.000
Atrial area of scar	96.70 ± 35.40 (n = 55)	92.60 ± 37.10 (n = 26)	98.10 ± 29.90 (n = 29)	0.869	97.40 ± 32.40 (n = 25)	95.15 ± 37.35	0.492
Health area (> 1.5 mV)	84.00 ± 31.80 (n = 55)	71.40 ± 41.50 (n = 26)	88.60 ± 29.65 (n = 29)	1.000	74.40 ± 39.80 (n = 25)	85.80 ± 30.78	0.495
TrZ area (0.5–1.5 mV)	5.60 ± 12.10 (n = 55)	**5.00 ± 12.22 (n = 26)**	**7.00 ± 12.75 (n = 29)**	**0.008**	5.60 ± 12.00 (n = 25)	6.30 ± 12.83	0.956
LVZ Area (< 0.5 mV)	1.60 ± 8.20 (n = 55)	1.75 ± 12.88 (n = 26)	1.20 ± 5.05 (n = 29)	0.487	0.70 ± 10.10 (n = 25)	1.65 ± 6.83	0.198

Galectin-3 (Gal-3) and H_2_O_2_ plasma levels in left atrium (LA) and peripheral vein in all men patients with AF and in relaxin-2 LA and peripheral vein subgroups according to the median relaxin-2 distribution. *Alpha defensin 3* (*DEFA3*) and *interleukin-6* (*IL-6*) expression levels in leucocytes from LA plasma in all patients with AF and in relaxin-2 LA and peripheral vein subgroups according to the median value of relaxin-2. Values are represented as median ± Interquartile Range (IQR) in pg/mL, ng/mL, arbitrary units (a.u.), μM or cm^2^. The exact *n* values are stated in the table depending on the subgroups and the molecules studied. Statistical analysis: Mann–Whitney U test. *p*-value for the difference between patient groups defined according to the median value of relaxin-2 distribution in LA and peripheral vein.

AF: atrial fibrillation; DEFA3: alpha defensin 3; Gal-3: galectin-3; IL-6: interleukin-6; IQR: interquartile range; LA: left atrium; LVZ: low-voltage zones; PV: pulmonary vein; TrZ: transition zone.

Significant values are in bold.

*p-value < 0.05, **p-value < 0.01, ***p-value < 0.001.

In women (n = 9), we could not detect significant differences (probably due to the small n) in Gal-3 and H_2_O_2_ plasma levels, nor *DEFA3* and *IL-6* mRNA expression in leucocytes from LA blood among the subgroups created according to the median value of relaxin-2 distribution in LA and peripheral vein (Table 6).

**Table 6:** Fibrotic, inflammatory and oxidative stress molecules, and markers of left-atrial voltage mapping in women patients with atrial fibrillation (AF) according to relaxin-2 subgroups.

	Women						
	All patients (n = 9)	Left atrium relaxin-2 plasma levels			Peripheral vein relaxin-2 plasma levels		
		≤ 21.43 ± 59.89 pg/mL (n = 5)	> 21.43 ± 59.89 pg/mL (n = 4)	*p*-value	≤ 92.24 ± 141.98 pg/mL (n = 4)	> 92.24 ± 141.98 pg/mL (n = 5)	*p*-value
LA Gal-3 (ng/mL), median ± IQR	15.09 ± 7.55	17.49 ± 8.89	14.90 ± 11.05	0.806	16.10 ± 11.50	15.09 ± 8.98	0.624
Peripheral vein Gal-3 (ng/mL), median ± IQR	14.30 ± 6.18	14.21 ± 7.51	14.57 ± 8.34	0.462	12.48 ± 7.56	14.30 ± 6.69	0.462
LA DEFA3 (a.u), median ± IQR	1.67 ± 0.10	1.73 ± 0.10	1.65 ± 0.06	0.110	1.66 ± 0.07	1.70 ± 0.12	0.389
LA IL-6 (a.u), median ± IQR	1.41 ± 0.04	1.41 ± 0.07	1.40 ± 0.03	0.327	1.39 ± 0.10	1.41 ± 0.03	0.462
LA H_2_O_2_ (μM), median ± IQR	0.15 ± 0.48	0.15 ± 0.58	0.14 ± 0.33	0.624	0.11 ± 0.47	0.21 ± 0.48	0.462
Peripheral vein H_2_O_2_ (μM), median ± IQR	0.06 ± 0.10	0.04 ± 0.11	0.09 ± 0.07	0.620	0.04 ± 0.07	0.12 ± 0.10	0.063
**LA voltage mapping (cm**^**2**^**), median ± IQR**							
Total area of scar	180.00 ± 79.45	170.30 ± 77.15	193.65 ± 93.75	1.000	175.15 ± 35.20	180.00 ± 126.90	1.000
Atrial area of scar	73.50 ± 48.10	73.50 ± 44.85	84.75 ± 55.97	1.000	74.25 ± 27.30	67.90 ± 71.90	1.000
Health area (> 1.5 mV)	54.10 ± 31.40	39.30 ± 20.80	69.95 ± 42.50	1.000	49.90 ± 33.82	57.90 ± 53.45	1.000
TrZ area (0.5–1.5 mV)	14.70 ± 16.70	14.70 ± 45.80	11.50 ± 15.75	1.000	17.15 ± 7.98	7.60 ± 48.40	0.444
LVZ area (< 0.5 mV)	2.00 ± 9.20	6.20 ± 11.40	0.45 ± 1.38	1.000	5.70 ± 9.55	0.50 ± 8.50	0.444

Galectin-3 (Gal-3) and H_2_O_2_ plasma levels in left atrium (LA) and peripheral vein in all women patients with AF and in relaxin-2 LA and peripheral vein subgroups according to the median relaxin-2 distribution. *Alpha defensin 3* (*DEFA3*) and *interleukin-6* (*IL-6*) expression levels in leucocytes from LA plasma in all patients with AF and in relaxin-2 LA and peripheral vein subgroups according to the median value of relaxin-2. Values are represented as median ± Interquartile Range (IQR) in pg/mL, ng/mL, arbitrary units (a.u.), μM or cm^2^. The exact *n* values are stated in the table depending on the subgroups and the molecule studied. Statistical analysis: Mann–Whitney U test. *p*-value for the difference between patient groups defined according to the median value of relaxin-2 distribution in LA and peripheral vein.

AF: atrial fibrillation; DEFA3: alpha defensin 3; Gal-3: galectin-3; IL-6: interleukin-6; IQR: interquartile range; LA: left atrium; LVZ: low-voltage zones; PV: pulmonary vein; TrZ: transition zone.

*p-value < 0.05, **p-value < 0.01, ***p-value < 0.001.

Additionally, LA and/or peripheral vein relaxin-2 plasma levels have shown significant correlations with Gal-3, *DEFA3*, *IL-6* or H_2_O_2_ in the whole population of AF patients subjected to study (Supplementary Table S8 online). Moreover, we have found a significant increase in LA (*p*-value = 0.001) and peripheral vein (*p*-value = 0.017) relaxin-2 plasma levels of AF patients with sinus rhythm compared to AF rhythm (Supplementary Fig. S1 online); together with a significant correlation between cardiac and/or peripheral relaxin-2 plasma levels with Gal-3, *DEFA3* and *IL-6* in AF patients with sinus rhythm, while these correlations were absent in AF patients with AF rhythm (Supplementary Table S9 online). We have also observed a significant increase in *IL-6* mRNA expression in leucocytes from LA blood (*p*-value = 0.001) and in H_2_O_2_ LA plasma levels (*p*-value = 0.019) in AF patients with AF rhythm (Supplementary Fig. S1 online).

### Relaxin-2 inhibits NHCF-A fibroblasts migration

**Figure 2:**
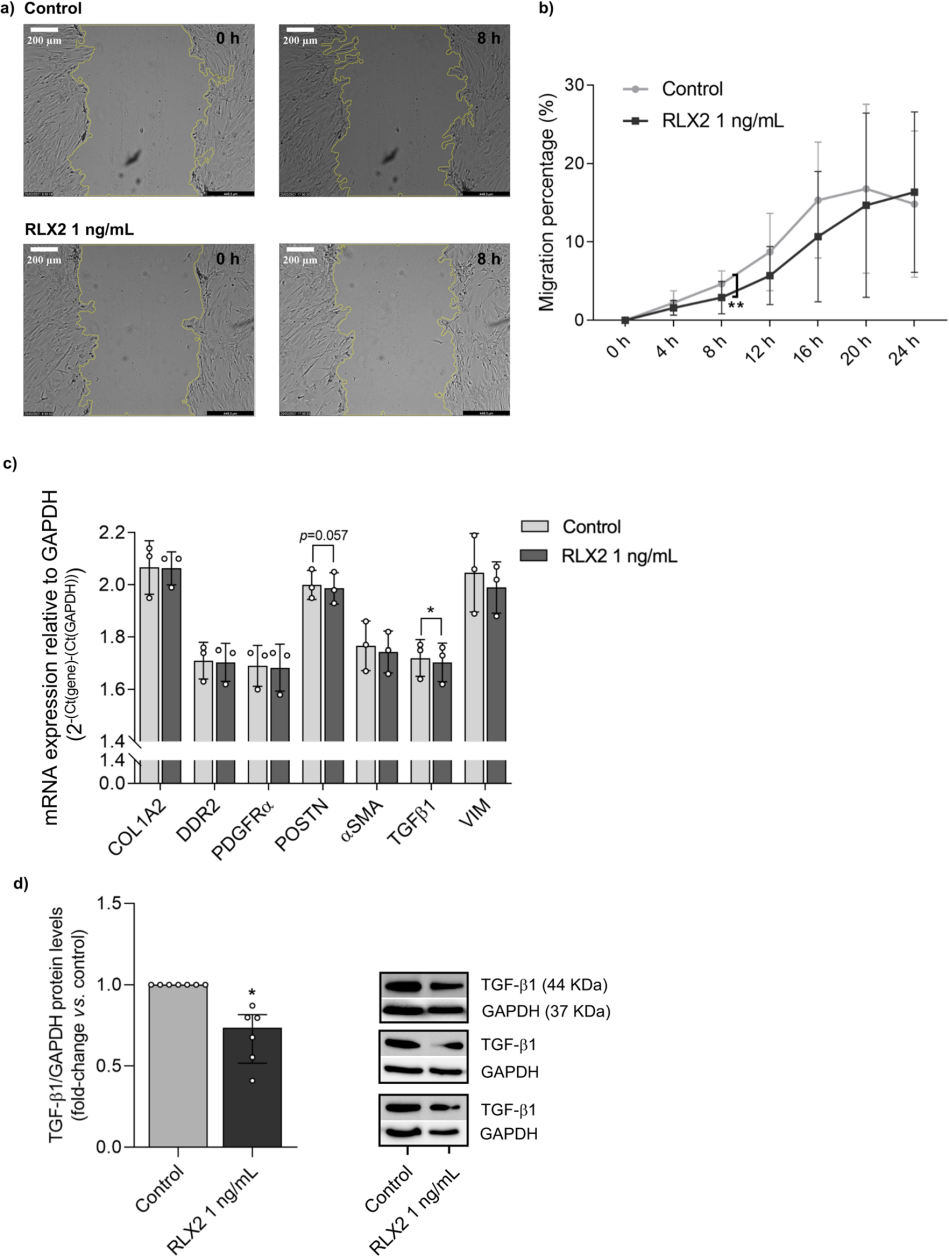
Effect of relaxin-2 in the migration ability of normal human atrial cardiac fibroblasts (NHCF-A) and in the expression of pro-fibrotic markers in NHCF-A. (**a**) Optical microscopy LasX DMI6000B Objective: 4X; (**b**) time course of migration assay for 4, 8, 12, 16, 20 and 24 h (n = 6 biological replicates × 4 technical replicates). (**c**) mRNA expression of selected genes after a 24-h treatment with RLX2 at 1 ng/mL (n = 3 biological replicates × 2 technical replicates). (**d**) TGF-β1 protein expression levels in NHCF-A after RLX2 treatment for 24 h (n = 6 biological replicates). Data are expressed as mean ± SD for (**b**,**c**), and as median ± interquartile range (IQR) for (**d**). Change among treatments were analysed by paired *t*-test (**b**,**c**) or Wilcoxon-signed rank test (**d**). *p-value < 0.05. Original uncropped blots are presented in Supplementary Figures S2 and S3 in the Supplementary Information file. αSMA: alpha smooth muscle actin; COL1A2: collagen type I alpha 2 chain; DDR2: discoidin domain receptor tyrosine kinase 2; GAPDH: glyceraldehyde-3-phosphate dehydrogenase; PDGFRα: platelet derived growth factor receptor alpha; POSTN: periostin; TGF-β1: transforming growth factor β1; VIM: vimentin.

In absence of FBS, RLX2 treatment showed a trend to reduce NHCF-A migration into the wound area. At 1 ng/mL, it affected significantly to wound-healing percentage with a peak of significance at 8 h after the wound was made (4.66 ± 0.74% in control vs. 2.91 ± 1.03% in RLX2 at 1 ng/mL, *p*-value = 0.008, n = 6 biological replicates × 4 technical replicates) (Fig. 2a, b).

### Relaxin-2 reduces profibrotic gene expression in NHCF-A

RLX2-treatment at 1 ng/mL for 24 h did not change the mRNA expression levels of *COL1A2*, *DDR2* and *PDGFRα* in NHCF-A cells. On the contrary, RLX2 tended to reduce mRNA expression levels of *αSMA*, *TGF-β1*, *POSTN* and *VIM*, reaching the statistical significance in *TGF-β1* (1.72 ± 0.07 arbitrary units (a.u.) in control vs. 1.70 ± 0.07 a.u. in RLX2, *p*-value = 0.038, n = 3 biological replicates × 2 technical replicates) in NHCF-A cells (Fig. 2c).

### Relaxin-2 decreases TGF-β1 protein levels in NHCF-A

We found that the treatment with 1 ng/mL of RLX2 for 24 h significantly decreased the protein levels of TGF-β1 (*p*-value = 0.031, n = 6 biological replicates) in NHCF-A cells (Fig. 2d).

## Discussion

During the last twenty years, a large number of clinical trials have been performed in order to evaluate the effect of relaxin-2 in different physiological and pathological circumstances^15,16^. At the same time, although numerous works have analyzed the plasma levels of relaxin-2 in patients with established cardiovascular diseases (including AF), it was not analyzed the potential relationship between relaxin-2 circulating levels and different risk and/or prognostic markers related to the metabolic state and body composition^8,9,17,18^. The promising use of relaxin-2 as a biomarker, risk predictor or therapeutic agent in cardiovascular disease is based on its probed capacity as a suppressor of inflammation and fibrosis, two physiopathological mechanisms of great relevance in AF and heart failure (HF)^19,20^.

### Relaxin-2 plasma levels in the peripheral and cardiac circulation of AF patients

In the present study, we have analyzed for the first time the concentrations of relaxin-2 in plasma from LA, and we have compared relaxin-2 plasma levels in the peripheral and cardiac circulation. Our AF population showed lower peripheral vein relaxin-2 plasma levels than those previously determined in plasma or serum from pregnant women^21^, but higher than relaxin-2 circulatory levels in men and menopausic women^18,22^, and similar to prior studies with AF patients^8,9^. Resembling the results reported by other authors in studies with HF patients^17,18^, our findings showed a high and variable concentration of relaxin-2 in plasma from AF patients, with only a 5.90% and 2.90% of patients having relaxin-2 levels below the detection limit in LA and peripheral vein, respectively. In men, we could hypothesize that the increase in peripheric relaxin-2 plasma levels compared with those detected in atria may be explained by the following reasons: (1) the secretion of relaxin-2 from peripheral tissues (atria, ventricles, kidney, lung, liver, spleen, pancreas, skin, vascular tissues), where it is highly expressed^23^ and/or (2) the fact that relaxin-2 produces its effects in the heart by acting directly on the relaxin family peptide receptor 1 (RXFP1), located mainly in the atria^23,24^; so the relaxin-2 available for detection by ELISA in atrial blood samples could be decreased by the high binding of the molecule to the highly expressed RXFP1 at this level. Moreover, our study has found for the first time a statistical difference in peripheral plasma levels of relaxin-2 related to gender. Women with AF showed a higher concentration of relaxin-2 in plasma from peripheral vein than men. However, it should be considered that, since patients were consecutively enrolled in the study, comparisons regarding plasmatic levels of relaxin-2 were made between 9 women and 59 men. In our study, we have found that relaxin-2 plasma levels in LA and peripheral vein are significantly increased in AF patients that are in sinus rhythm at the moment of the procedure (which implies less severe disease). In this way, we could hypothesize that increased LA and peripheral relaxin-2 plasma levels in AF patients who presented sinus rhythm in the study, could be exerting the multiple beneficial cardioprotective effects during pathological events (AF in this case) that have been described for this molecule, and that include suppression of arrhythmia and inflammation, reversal of fibrosis or amelioration of oxidative stress^19,20^. Until now, only two works have reported relaxin-2 circulatory levels in patients with AF^8,9^, but in none of them has been described an association between relaxin-2 levels and the rhythm of the patients, except for the work of Zhou et al. (2016), which included control patients with sinus rhythm (without AF, and for this reason not comparable to our study) in which serum relaxin-2 levels were significantly lower than in both paroxysmal and persistent AF patients^8^. The increase in *IL-6* mRNA expression in leucocytes from LA blood, and the increase in LA H_2_O_2_ plasma levels in AF patients with AF rhythm could be related to tissular damage in these patients with a more severe AF^25^.

### Association between LA and peripheral vein plasma levels of relaxin-2 in AF patients with molecules implicated in fibrosis, inflammation and oxidative stress

Recent research findings in animal models and human clinical trials have highlighted a pivotal role of fibrosis, inflammation and oxidative stress on the risk, onset, progression and maintenance of AF^14,26,27^. Therefore, we decided to study the association among plasma levels of relaxin-2 in LA and peripheral vein of AF patients with molecules implicated in fibrosis, inflammation and oxidative stress, which could be useful for AF risk prediction and could help to identify possible pathways or markers involved in the onset of AF, as well as to evaluate promising therapeutic strategies for AF, such as relaxin-2 or serelaxin treatment.

#### Fibrosis

Regarding fibrosis, a common pathologic insult in AF, our findings showed that AF patients with high levels of relaxin-2 in plasma from peripheral vein also had high levels of Gal-3 in plasma from peripheral vein. Gal-3 is a beta-galactoside-binding lectin that presents an increased expression in fibrotic tissues and acts as a regulator of fibrosis and inflammation, being considered an emerging biomarker for cardiovascular diseases^28,29^, especially for the cardiac remodelling that takes place in AF patients^30^. In fact, some authors have suggested that an increase in the circulatory levels of Gal-3 could be associated with a higher risk for developing AF^31^, although others do not support a role of Gal-3 in AF risk prediction after accounting for other traditional clinical AF risk factors^32^. Despite circulatory Gal-3 levels are augmented in AF patients undergoing radiofrequency catheter ablation, Gal-3 is not useful for predicting rhythm outcome, because that increase could be driven by cardiometabolic comorbidities and not by heart rhythm, so further studies are necessary to establish if Gal-3 could predict recurrence^33^. Relaxin-2 possesses a robust antifibrotic effect in the cardiovascular system^1,2,6^, and similarly to our results regarding Gal-3, it has been determined that AF patients with high relaxin-2 serum levels also showed an increase in serum levels of several fibrotic and inflammatory biomarkers such as TGF-β, PICP and TNF-α^8^. Taken together, all these evidence suggest that relaxin-2 is tightly associated with the mechanisms of fibrosis, and with different fibrotic biomarkers, suggesting that the endogenous synthesis of relaxin-2 could be increased as a compensatory mechanism in response to a fibrotic stimulus in the cardiac tissue. Furthermore, our results demonstrate for the first time that relaxin-2 treatment inhibits normal human atrial cardiac fibroblasts migration (which is in concordance with previous findings in rat cardiac fibroblasts^34^) and, additionally, reduces *TGF-β1* mRNA and protein expression in our cells, in agreement with the proposed role for relaxin-2 in the disruption of the pro-fibrotic TGF-β1/IL-1β axis^35^, underpinning a potential therapeutic use of relaxin-2 for the inhibition of cardiac fibrosis, which could be suitable for AF management.

#### Inflammation

Inflammation could stimulate AF development, but could be also induced during AF existence; and therefore, atrial cardiomyocyte injury during AF may cause an inflammatory response and remodelling^27^. In fact, previous works have described an increase in pro-inflammatory cytokine levels in AF patients in comparison with healthy volunteers, a scenario that could be related to atrial fibrosis and to cardiomyocyte damage and apoptosis^36^. Additionally, the presence of inflammation in the heart or systemic circulation can predict the onset and recurrence of AF^27^. In the present study, we found that AF patients with high circulatory levels of relaxin-2 showed a decrease in the gene expression of two inflammatory markers (DEFA3 and IL-6) in leucocytes from LA plasma. Little is known about the role in AF of DEFA3, a protein secreted by epicardial fat which is involved in inflammation, and whose plasma levels have been found decreased in patients with post-surgery AF^37^, being considered as a likely inflammatory risk and prognostic predictor of cardiovascular disease^37–39^. IL-6 is a cytokine capable of inducing inflammatory cell adhesion and aggregation, together with the activation of acute-phase reactive proteins (such as fibrinogen or C-reactive protein), causing atrial muscle inflammation, left ventricle hypertrophy, ventricle stiffness, increase in collagen content, atrial fibrosis and AF onset^27,40^. Moreover, AF patients present high plasma levels of IL-6, which is supposed to participate in the development and maintenance of AF^27^. In fact, elevated plasma levels of IL-6 are directly correlated with LA size, AF duration, and AF recurrence after radiofrequency catheter ablation^41–43^. The widely known anti-inflammatory role of relaxin-2 on cardiac tissue (including the decrease in pro-inflammatory cytokines like IL-6, IL-1β, TNF-α and monocyte chemoattractant protein-1 (MCP-1), or the reduction in macrophage and neutrophil infiltration)^3,44–46^, in addition to our findings presented here, may reinforce the hypothesis of a role for relaxin-2 as a modulator of inflammation and atrial remodelling.

#### Oxidative stress

Oxidative stress is another important mechanism that contributes to the onset of AF through, for example, the increase in O_2_^–^ via NAD(P)H oxidase action in human atria^14,47,48^. Oxidative stress occurrence could alter cardiac electrophysiological characteristics, regulate calcium handling on cardiomyocytes, stimulate inflammation, increase fibroblasts proliferation in atria and contribute to atrial fibrosis and AF progress^47,49,50^. Also, another factor linked to AF development is an increase in H_2_O_2_ levels, that are in close relationship with structural remodelling and with atrial fibrosis, and which could modify the electrophysiological features of pulmonary veins and LA, as well as atrial calcium homeostasis, provoking AF occurrence^51–53^. Our findings have shown that men with AF and with higher plasma levels of relaxin-2 in peripheral vein presented a reduction in circulatory levels of H_2_O_2_ in peripheral vein. To notice, several previous preclinical and clinical studies have described that relaxin-2 treatment could induce an antioxidant effect (decreasing O_2_^–^, reactive oxygen species (ROS), lactate dehydrogenase (LDH), malondialdehyde (MDA) and uric acid, while increasing catalase, glutathione peroxidase (GPX) and glutathione (GSH)) and could reduce H_2_O_2_ levels in the cardiovascular system^54–57^. All those evidence, in addition to our present findings, could lead to hypothesize that endogenous relaxin-2 could modulate H_2_O_2_ plasma levels in response to AF; an aspect of relevance for the potential use of this hormone as a therapeutic strategy in AF management^58^.

### Conclusions

The results of this work may contribute to clarify the role of relaxin-2 in AF physiopathology, providing new insights about the functions of this hormone in atrial structural remodelling. Likewise, our findings infer that relaxin-2 plasma levels in AF patients could be useful as a biomarker in the context of this pathology.

All clinical trials with relaxin-2 in patients with fibrotic diseases have given until now inconclusive results, but a recent meta-analysis of the relaxin-2 effects on AHF has come to the conclusion that relaxin-2 administration significantly reduces the risk of worsening of symptoms and ameliorates renal function markers^59^, what points to the necessity of additional accurately-designed future clinical investigations. The results of our work, demonstrating the antifibrotic effects of relaxin-2, especially the ability to interfere with TGF-β1 signalling and to reduce fibroblast recruitment and activation, may help to provide useful data to investigate some future opportunities for the therapeutic use of relaxin-2 in fibrotic diseases, as HF and/or AF.

## Limitations of the study

The main limitation of this study is the different number of men and women that participated in the study, which is mainly due to the fact that patients were recruited consecutively from October 2016 to October 2017. Atrial fibrosis from patients was not quantified by imaging technique analysis, which is more accurate than Gal-3 determination and/or voltage mapping. The patient recruitment was performed in a single centre. Since we needed LA plasma to test relaxin-2 levels, the recruitment of the patients was performed before pulmonary vein radiofrequency catheter ablation, which implies that we have no control healthy subjects for comparison. Blood samples from the patients were only collected before pulmonary vein radiofrequency catheter ablation, and future studies should include blood samples and image analysis during the follow-up of the patients. The in vitro model with NHCF-A cells constitutes a preclinical approach for the study of the physiopathological mechanisms of AF, but further additional studies are needed with, for example, animal models, in order to explain the antifibrotic mechanisms of relaxin-2 from a clinical perspective.

## Methods

### Materials

All reagents were obtained from Sigma-Aldrich (MO, USA) unless otherwise stated. Recombinant human luteal relaxin-2 (RLX2) was kindly provided by Dr. Mario Bigazzi and Dr. Daniele Bani (Foundation for Research on Relaxin in Cardiovascular and other Diseases in Prosperius Institute and Department of Experimental & Clinical Medicine in the Univer-sity of Florence, IT).

### Study population

The population of study was composed by 68 consecutive Caucasian patients (59 men and 9 women) with paroxysmal, persistent or long-standing persistent AF, which were subjected to pulmonary vein radiofrequency catheter ablation in the Cardiovascular Area of the University Clinical Hospital of Santiago de Compostela. The exclusion criteria were age under 18 years, pregnancy, and any latent infectious condition. None of the 68 AF patients included in the study presented a history of previous AF ablation.

### Blood sample collection

During catheter ablation procedure (after 8 h of fasting), immediately after the transseptal puncture and preceding to heparin administration, blood samples were obtained from the left atrium (LA) through the transseptal sheath, and simultaneously, peripheral vein blood samples were obtained from the right femoral vein. LA and peripheral vein blood samples were collected in EDTA tubes and were centrifuged with or without aprotinin (PanReac AppliChem ITW Reagents, NL) at 2000 g for 15 min and 4 °C. Electrical cardioversion was performed after the blood extraction (in order to avoid any change that could lead to a modification in the study biomarkers) and before the left atrial reconstruction and ablation. Our workflow was (1) to obtain the femoral accesses; (2) transseptal puncture; (3) once in the left atrium, to draw the blood from the transseptal sheath and simultaneously from the right femoral vein; (4) to perform the electrical cardioversion; (5) once in sinus rhythm, the left atrial map is performed and the pulmonary vein targeted.

### Left-atrial voltage mapping

Patients underwent point-by-point radiofrequency catheter ablation (SmartTouch, Biosense Inc., USA). The procedural endpoint was ipsilateral pulmonary vein isolation (PVI).

Assessment of LA surface area and LA fibrosis was based on bipolar voltage map, which was created simultaneously with LA surface reconstruction, guided by a three-dimensional electroanatomical mapping system (CARTO3, Biosense Webster, USA) using a multipolar mapping catheter (PentaRay, Biosense Webster, USA). Adequate quality of the acquired voltage points was established according to CONFIDENSE module after respiratory compensation. This is a continuous mapping software with automated data acquisition when set-criteria are met, among them: (1) tissue proximity indication (proximity-based filtering of points acquired with the PentaRay); (2) wavefront annotation (an automated annotation algorithm that incorporates both unipolar and bipolar signals); (3) map consistency (system identifies outlying points when certain criteria are met; (4) position stability filter (ensures catheter position is consistent with previous location, settled in < 5 mm); (5) cycle length stability (keeps data collected within a range of predefined cycle lengths, settled in 10% over the average). A minimum number of points were requested (> 1000) and the density fill threshold remained constant at ≤ 5 mm.

The LA anatomical maps and all acquired points were meticulously manually reviewed and annotated offline. Three different cut-offs were used for low-voltage zones (LVZ), according to the underlying rhythm. In sinus rhythm mapping the LVZ cut-off was < 0.5 mV and TrZ range was between 0.5 and 1 mV. If mapping was in AF, LVZ cut-off was < 0.24 mV and in atrial flutter (AFL) < 0.3 mV. Low voltage zone was identified as an area of at least 1 cm^2^ in area containing ≥ 3 neighbouring points with ≤ 10 mm distance^60^. The LVZ and transition zone (TrZ) area were measured by manually encircling the area with a measurement tool and was expressed in cm^2^. Burden was calculated as the percentage of total left atrial surface area excluding the pulmonary vein (PV) ostia and mitral valve area. All patients underwent ipsilateral wide area circumferential PVI with the use of contact force (CF) sensing irrigated tip ablation catheter (Smart-Touch®, Biosense Webster, Diamond Bar, USA) and automatic ablation annotation module (Visi-Tag®, Biosense Webster, Diamond Bar, USA).

### Biomarker measurement

#### Relaxin-2 plasma determination

Relaxin-2 plasma levels were measured using a commercial enzyme-linked immunosorbent assay (ELISA) kit (Cat# K9210, Immunodiagnostik AG, DE), according to manufacturers’ instructions. Results are the average of duplicate measurements. The detection limit of this assay is 0.50 pg/mL. The intra-assay coefficients of variation were 4.7% for 34.4 pg/mL (n = 20), 2.5% for 99.6 pg/mL (n = 20) and 2.3% for 206.0 pg/mL (n = 20). The inter-assay coefficients of variation were 10.2% for 40.8 pg/mL (n = 42), 6.3% for 112.0 pg/mL (n = 42) and 5.5% for 220 pg/mL (n = 42). If relaxin-2 levels were below the detection limit, they were fixed at 0.4 pg/mL for data analysis, as previously reported^17^.

#### Galectin-3 plasma measurement

Galectin-3 plasma levels were determined using a commercial ELISA kit (Cat# BMS279/4TEN, eBioscience, Thermo Fisher Scientific, AT), according to the manufacturers’ protocols. Results are the average of duplicate measurements. The detection limit of this assay is 0.29 ng/mL. The intra-assay and inter-assay coefficients of variation were 7.5% and 5.4%, respectively.

#### IL-6 and alpha-defensin 3 mRNA expression levels in leucocytes

After centrifugation, atrial blood leucocytes were collected from the blood cells white layer, which was transferred into another tube for lysing erythrocytes with 155 mM NH_4_Cl. Afterwards, cells were precipitated and washed with saline solution and centrifugated for 5 min. At the end, cells were lysed with RLT (Qiagen, GE), RNA was isolated by Allprep RNA/protein kit and complementary DNA (cDNA) was synthesized by Maxima Reverse Transcriptase activity (Thermo Scientific, USA). After retrotranscription, 2 μl of cDNA was used for *interleukin-6* (*IL-6*) and *alpha-defensin 3* (*DEFA3*) amplification using the FastStart SYBR Green Master (Hoffmann-La Roche, CH). The primers *IL-6* (Accession number: NM_001371096.1, F-TGAGGTGCCCATGCTACATTT, R-GTGGCTGCAGGACATGACAA)^61^, *DEFA3* (Accession number: NM_005217, F-TCCCAGAAGTGGTTGTTTCC, R-CAGAATGCCCAGAGTCTTCC)^62^, and *ACTB* (*β-actin*) (Accession number: NM_001101.5, F-TTCTGACCCATGCCCACCAT; R-ATGGATGATGATATCGCCGCGCTC)^63^ were amplified at 40 cycles (95 °C for 30 s, 60 °C for 60 s) in a Stratagene Mx3005P Real-Time PCR Machine (Agilent Technologies, USA). The cycle threshold (Ct) values of *IL-6* and *DEFA3* were normalized by the Ct values of *β-actin* (ΔCt) of control and RLX2 group (2^-ΔCt^; hereafter referred to as gene expression levels).

#### H_2_O_2_ plasma levels

LA and peripheral vein plasma levels of H_2_O_2_ were determined by an enzymatic assay that utilizes the chromogenic Fe^3+^ − xylenol orange reaction with a detection range of 0.2–30 μM (Merck, DEU).

### Cell culture

Normal human atrial cardiac fibroblasts (NHCF-A) were obtained from Lonza (Lonza Biologics, CH, RRID:SCR_000377), and were thawed, seeded, and maintained following manufacture’s protocol. The cells were incubated in a humidified atmosphere of 5% CO_2_ at 37℃, media was renewed every 2–3 days, and cells were passaged when they reached approximately 90% confluence with trypsin–EDTA solution; 25 cm^2^ flasks were used for primary cell culture maintenance. NHCF-A cells were seeded in 6 or 24 multiwell plates for cell treatments with RLX2. NHCF-A primary culture cells showed mRNA expression of *fibronectin extra domain A* (*FN-EDA*) (2.73 ± 0.37 in control; 3.20 ± 0.35 in RLX2 at 1 ng/mL, n = 3) and *myosin heavy chain 10* (*MYH10*) (2.96 × 10^–2^ ± 6.63 × 10^–1^ in control; 2.45 × 10^–2^ ± 4.22 × 10^–1^ in RLX2 at 1 ng/mL, n = 3). Having into account these data, we cannot exclude a partial transition from cardiac fibroblast to myofibroblast phenotype in our cell culture system. NHCF-A were isolated from normal, adult heart tissue.

### In vitro scratch wound-healing assay

To perform the wound-healing assay, NHCF-A were seeded for the at approximately 1 × 10^5^ per well in 24-multiwell plates. Cells were serum-deprived for 8 h prior to the beginning of the experiment. Using a plastic pipette tip, a wound field was created with a defined gap of 1 mm in each well, and cells were then treated with RLX2 at 1 or 10 ng/mL, or vehicle (phosphate-buffered saline (PBS)) (n = 6 biological replicates × 4 technical replicates), as previously described^35,54,64^, without fetal bovine serum (FBS) to inhibit cell proliferation and to allow cell migration. Random fields were photographed with a Leica DMI6000B Automated/Motorized Inverted Microscope (Leica Microsystems, DE) during the time-course procedure. The wound areas tracing the cell-free area were quantified at different times (0, 4, 8, 12, 16 and 24 h) with the Fiji ImageJ software. Wound areas were relativized with respect to the area of the initial time (t0). Results are expressed as a percentage of migration:

$$Migration \;percentage\; \left( \% \right) = \frac{{\left( {A0 - Ax} \right)}}{A0} \times 100$$

being A0 the area of wound measured at 0 h and Ax the area of the wound measured at the different times (4, 8, 12, 16 and 24 h). The experiment was performed in NHCF-A between 4 and 6 passages (n = 6 biological replicates).

### mRNA expression of the main characteristic genes of NHCF-A cells after RLX2 treatment

NHCF-A were seeded in 24-multiwell plates at the same confluence as for the wound-healing assays. After serum-deprivation for 8 h, cells were treated with RLX2 at a dose of 1 ng/mL^35,54,64^, or with vehicle (PBS) (condition = 3 biological replicates × 4 technical replicates) for 24 h. At the end of the process, cells were lysed for the determination of *collagen type I alpha 2 chain* (*COL1A2*), *discoidin domain receptor tyrosine kinase 2* (*DDR2*), *platelet derived growth factor receptor alpha* (*PDGFRα*), *periostin* (*POSTN*), *alpha smooth muscle actin* (*αSMA), transforming growth factor β1* (*TGF-β1*), *vimentin* (*VIM*) and *glyceraldehyde-3-phosphate dehydrogenase* (*GAPDH*) levels. Moreover, mRNA expression of *fibronectin extra domain A* (*FN-EDA*) and *myosin heavy chain 10* (*MYH10*) were determined in order to phenotype NHCF-A cells. Thus, RNA was isolated with the High Pure RNA Isolation Kit (Hoffmann‐La Roche, CH), following the manufacture’s protocol, and cDNA was synthesized using the Maxima First Strand cDNA Synthesis Kit (Thermo Fisher Scientific, USA). Real-time PCR with the primers *COL1A2*, *DDR2*, *PDGFRα*, *POSTN*, *αSMA*, *TGF-β1*, *VIM*, *FN-EDA* and *MYH10* was performed for quantifying the mRNA expression levels with respect to GAPDH levels as it was previously described (*COL1A2* (Accession number: NM_000089.3)^65^: F‐TCGCACATGCCGTGACTTG; R‐GATAGCATCCATAGTGCATCCTTG, *DDR2* (Accession number: NM_001014796.3)^66^: F‐AACGAGAGTGCCACCAATGGCT; R‐ACTCACTGGCTTCAGAGCGGAA, *PDGFRα* (Accession number: NM_001347830.2)^67^: F‐GACTTTCGCCAAAGTGGAGGAG; R‐AGCCACCGTGAGTTCAGAACGC, *POSTN* (Accession number: NM_001135935.2)^67^: F‐CGTTGCTCTCCAAACCTCTA; R‐TGCCCAGCAGTTTTGCCCAT, *αSMA* (Accession number: NM_001613.4)^68^: F‐CCGACCGAATGCAGAAGGA; R‐ACAGAGTATTTGCGCTCCGAA, *TGF-β1* (Accession number: NM_000660.7)^69^: F‐TACCTGAACCCGTGTTGCTCTC; R-GTTGCTGAGGTATCGCCAGGAA, *VIM* (Accession number: NM_003380.5)^70^: F‐AGGCAAAGCAGGAGTCCACTGA; R-ATCTGGCGTTCCAGGGACTCAT, *FN-EDA* (Accession number: NM_002026.4)^71^: F-*CCAGTCCACAGCTATTCCTG*; R-ACAACCACGGATGAGCTG, *MYH10* (Accession number: NM_001256095.2)^72^: F- *TCCCGCTGGAGTTTACGC*; R-GCAGGAAGCCAAGGAACG and *GAPDH* (Accession number: NM_001357943.2)^73^: F‐CATGTTCGTCATGGGGTGAACCA; R‐AGTGATGGCATGGACTGTGGTCAT).

### Western blotting

NHCF-A were seeded in 6-multiwell plates and after serum-deprivation for 8 h, cells were treated with RLX2 using a dose of 1 ng/mL or vehicle (PBS) for 24 h (n = 6 biological replicates)^35,54,64^. NHCF-A were lysed and subjected to SDS-PAGE/Western blotting as previously described^74^, using primary antibodies against transforming growth factor-β1 (TGF-β1) (1:1000, Cat# ab179695, abcam, GB) according to manufacturer instructions. We employed mouse anti-rabbit IgG-HRP (1:1000, Cat# sc-2357, Santa Cruz Biotechnology, Inc., USA) as secondary antibody. Visualization of protein band intensities was determined by chemiluminescence using Clarity Western ECL Substrate (Bio-Rad Laboratories, Inc., USA) and a ChemiDoc MP Imaging System (Bio-Rad Laboratories, Inc., USA) and normalized using glyceraldehyde-3-phosphate dehydrogenase (GAPDH) (1:1000, Cat# G9545, Sigma-Aldrich, USA) when quantified.

### Statistical analysis

For descriptive analysis of the clinical data, categorical variables are expressed as counts and proportions, while continuous variables are expressed as median ± interquartile range (IQR) and exact *n* values for each variable are depicted in tables. Patients were divided according to the median value of relaxin-2 distribution in LA and peripheral vein, and the subgroups were compared regarding clinical and analytical parameters, as well as biomarkers measurements. Comparison between groups was performed applying Fisher’s exact test for categorical variables, and Mann–Whitney U-test for non-normally distributed continuous variables. Normally distributed variables were expressed as mean ± Standard Deviation (SD), and non-normally distributed variables as median ± IQR. Correlation tests between biomarkers and relaxin-2 plasma levels were performed with the Spearman bivariate correlation analysis. Logistic regression models were performed in order to test which variables could be added to the set of risk factors for AF (age, body mass index (BMI) and arterial hypertension (AHT)), with the aim of significantly increase the predictive ability of that set of covariates. For in vitro experimental analysis, data are expressed as mean ± SD or median ± IQR of at least three individual measurements per group. Normality test used were Kolmogorov–Smirnov. Comparisons between experimental groups and controls were performed with the paired t-test or the Wilcoxon-signed rank test. Statistical analysis was performed using GraphPad Prism 5 (GraphPad Software Inc., USA) and IBM SPSS Statistics 20.0 (IBM, USA). The analysis of the measurements was carried out by a different scientist in order to avoid observer bias. A *p*-value < 0.05 was considered significant.

### Ethics approval statement

The study protocol follows the ethical guidelines of Declaration of Helsinki and the Medical Research Council's guidance and was reviewed and approved by the Galician Clinical Research Ethics Committee (Approval number 2014/356). All the patients that participated in the study signed the written informed consent.

## Supplementary Information

Supplementary Information.

## Data Availability

The datasets used and/or analyzed during the current study are available from the corresponding author upon reasonable request.

## References

[1] Samuel CS (2004). Relaxin modulates cardiac fibroblast proliferation, differentiation, and collagen production and reverses cardiac fibrosis in vivo. Endocrinology.

[2] Wang C (2020). The anti-fibrotic actions of relaxin are mediated through AT 2 R-associated protein phosphatases via RXFP1-AT 2 R functional crosstalk in human cardiac myofibroblasts. FASEB J..

[3] Beiert T (2018). Chronic lower-dose relaxin administration protects from arrhythmia in experimental myocardial infarction due to anti-inflammatory and anti-fibrotic properties. Int. J. Cardiol..

[4] Parikh A (2013). Relaxin suppresses atrial fibrillation by reversing fibrosis and myocyte hypertrophy and increasing conduction velocity and sodium current in spontaneously hypertensive rat hearts. Circ. Res..

[5] Henry BL (2016). Relaxin suppresses atrial fibrillation in aged rats by reversing fibrosis and upregulating Na+ channels. Heart Rhythm.

[6] Sassoli C (2013). Relaxin prevents cardiac fibroblast-myofibroblast transition via notch-1-mediated inhibition of TGF-β/Smad3 signaling. PLoS ONE.

[7] Piedras-Rentería ES, Sherwood OD, Best PM (1997). Effects of relaxin on rat atrial myocytes. I. Inhibition of I(to) via PKA-dependent phosphorylation. Am. J. Physiol..

[8] Zhou H (2016). Relaxin level in patients with atrial fibrillation and association with heart failure occurrence. Medicine (Baltimore).

[9] Qu X (2019). Serum relaxin level predicts recurrence of atrial fibrillation after radiofrequency catheter ablation. Heart Vessels.

[10] Teerlink JR (2013). Serelaxin, recombinant human relaxin-2, for treatment of acute heart failure (RELAX-AHF): A randomised, placebo-controlled trial. Lancet (London, England).

[11] Teerlink JR (2017). Serelaxin in addition to standard therapy in acute heart failure: Rationale and design of the RELAX-AHF-2 study. Eur. J. Heart Fail..

[12] Filippatos G (2017). Serelaxin in acute heart failure patients with and without atrial fibrillation: A secondary analysis of the RELAX-AHF trial. Clin. Res. Cardiol..

[13] Zhao Z, Ng CY, Liu T, Li H, Li G (2014). Relaxin as novel strategy in the management of atrial fibrillation: Potential roles and future perspectives. Int. J. Cardiol..

[14] Sagris M (2021). Atrial fibrillation: Pathogenesis, predisposing factors, and genetics. Int. J. Mol. Sci..

[15] Unemori E (2017). Serelaxin in clinical development: Past, present and future. Br. J. Pharmacol..

[16] Gifford FJ (2020). A phase 2 randomised controlled trial of serelaxin to lower portal pressure in cirrhosis (STOPP). Trials.

[17] Pintalhao M (2017). Relaxin serum levels in acute heart failure are associated with pulmonary hypertension and right heart overload. Eur. J. Heart Fail..

[18] Han L (2017). Combined assessment of relaxin and B-type natriuretic peptide improves diagnostic value in patients with congestive heart failure. Am. J. Med. Sci..

[19] Martin B, Romero G, Salama G (2019). Cardioprotective actions of relaxin. Mol. Cell. Endocrinol..

[20] Bani D (2020). Recombinant human H2 relaxin (serelaxin) as a cardiovascular drug: Aiming at the right target. Drug Discov. Today.

[21] Johnson MR, Abbas AA, Allman ACJ, Nicolaides KH, Lightman SL (1994). The regulation of plasma relaxin levels during human pregnancy. J. Endocrinol..

[22] Giordano N (2005). Serum relaxin in systemic sclerosis. J. Rheumatol..

[23] Bathgate RAD (2013). Relaxin family peptides and their receptors. Physiol. Rev..

[24] Dschietzig T (2011). The positive inotropic effect of relaxin-2 in human atrial myocardium is preserved in end-stage heart failure: Role of G(i)-phosphoinositide-3 kinase signaling. J. Card. Fail..

[25] Li Z (2022). Long atrial fibrillation duration and early recurrence are reliable predictors of late recurrence after radiofrequency catheter ablation. Front. Cardiovasc. Med..

[26] Sohns C, Marrouche NF (2020). Atrial fibrillation and cardiac fibrosis. Eur. Heart J..

[27] Hu Y-F, Chen Y-J, Lin Y-J, Chen S-A (2015). Inflammation and the pathogenesis of atrial fibrillation. Nat. Rev. Cardiol..

[28] Clementy N (2018). Galectin-3 in atrial fibrillation: Mechanisms and therapeutic implications. Int. J. Mol. Sci..

[29] Yu L (2013). Genetic and pharmacological inhibition of galectin-3 prevents cardiac remodeling by interfering with myocardial fibrogenesis. Circ. Heart Fail..

[30] Hernández-Romero D (2017). Galectin-3 as a marker of interstitial atrial remodelling involved in atrial fibrillation. Sci. Rep..

[31] Fashanu OE (2017). Galectin-3 and incidence of atrial fibrillation: The Atherosclerosis Risk in Communities (ARIC) study. Am. Heart J..

[32] Ho JE (2014). Galectin 3 and incident atrial fibrillation in the community. Am. Heart J..

[33] Clementy N (2016). Serum galectin-3 levels predict recurrences after ablation of atrial fibrillation. Sci. Rep..

[34] Wu X, Wang H, Wang Y, Shen H, Tan Y (2018). Serelaxin inhibits differentiation and fibrotic behaviors of cardiac fibroblasts by suppressing ALK-5/Smad2/3 signaling pathway. Exp. Cell Res..

[35] Cáceres FT, Gaspari TA, Samuel CS, Pinar AA (2019). Serelaxin inhibits the profibrotic TGF-β1/IL-1β axis by targeting TLR-4 and the NLRP3 inflammasome in cardiac myofibroblasts. FASEB J..

[36] Pan J (2018). Inflammatory cytokines in cardiac pacing patients with atrial fibrillation and asymptomatic atrial fibrillation. Panminerva Med..

[37] Couselo-Seijas M (2019). Cholinergic activity regulates the secretome of epicardial adipose tissue: Association with atrial fibrillation. J. Cell. Physiol..

[38] Christensen HM (2012). α-Defensins and outcome in patients with chronic heart failure. Eur. J. Heart Fail..

[39] Maneerat Y, Prasongsukarn K, Benjathummarak S, Dechkhajorn W, Chaisri U (2016). Increased alpha-defensin expression is associated with risk of coronary heart disease: A feasible predictive inflammatory biomarker of coronary heart disease in hyperlipidemia patients. Lipids Health Dis..

[40] Meléndez GC (2010). Interleukin 6 mediates myocardial fibrosis, concentric hypertrophy, and diastolic dysfunction in rats. Hypertension.

[41] Leftheriotis DI (2009). The predictive value of inflammatory and oxidative markers following the successful cardioversion of persistent lone atrial fibrillation. Int. J. Cardiol..

[42] Henningsen KMA (2009). Prognostic impact of hs-CRP and IL-6 in patients undergoing radiofrequency catheter ablation for atrial fibrillation. Scand. Cardiovasc. J..

[43] Psychari SN (2005). Relation of elevated C-reactive protein and interleukin-6 levels to left atrial size and duration of episodes in patients with atrial fibrillation. Am. J. Cardiol..

[44] Perna A (2005). Novel drug development opportunity for relaxin in acute myocardial infarction: Evidences from a swine model. FASEB J..

[45] Sanchez-Mas J (2017). Early anti-inflammatory and pro-angiogenic myocardial effects of intravenous serelaxin infusion for 72 H in an experimental rat model of acute myocardial infarction. J. Cardiovasc. Transl. Res..

[46] Masini E (2004). Relaxin inhibits the activation of human neutrophils: Involvement of the nitric oxide pathway. Endocrinology.

[47] Dudley SC (2005). Atrial fibrillation increases production of superoxide by the left atrium and left atrial appendage: Role of the NADPH and xanthine oxidases. Circulation.

[48] Kim YM (2005). A myocardial Nox2 containing NAD(P)H oxidase contributes to oxidative stress in human atrial fibrillation. Circ. Res..

[49] Xie W (2015). Mitochondrial oxidative stress promotes atrial fibrillation. Sci. Rep..

[50] Liang X (2018). Reactive oxygen species mediated oxidative stress links diabetes and atrial fibrillation. Mol. Med. Rep..

[51] Zhang J (2012). NOX4-dependent hydrogen peroxide overproduction in human atrial fibrillation and HL-1 atrial cells: Relationship to hypertension. Front. Physiol..

[52] Morita N (2009). Increased susceptibility of aged hearts to ventricular fibrillation during oxidative stress. Am. J. Physiol. Circ. Physiol..

[53] Lin YK (2010). Oxidative stress on pulmonary vein and left atrium arrhythmogenesis. Circ. J..

[54] Nistri S, Fiorillo C, Becatti M, Bani D (2020). Human relaxin-2 (serelaxin) attenuates oxidative stress in cardiac muscle cells exposed in vitro to hypoxia-reoxygenation. Evidence for the involvement of reduced glutathione up-regulation. Antioxidants (Basel, Switzerland).

[55] Wei X (2018). Relaxin ameliorates high glucose-induced cardiomyocyte hypertrophy and apoptosis via the Notch1 pathway. Exp. Ther. Med..

[56] Sasser JM, Cunningham MW, Baylis C (2014). Serelaxin reduces oxidative stress and asymmetric dimethylarginine in angiotensin II-induced hypertension. Am. J. Physiol. Ren. Physiol..

[57] Metra M (2013). Effect of serelaxin on cardiac, renal, and hepatic biomarkers in the relaxin in acute heart failure (RELAX-AHF) development program: Correlation with outcomes. J. Am. Coll. Cardiol..

[58] Hanafy DA (2013). Different effects of dronedarone and amiodarone on pulmonary vein electrophysiology, mechanical properties and H2O2-induced arrhythmogenicity. Eur. J. Pharmacol..

[59] Teerlink JR (2020). Effects of serelaxin in patients admitted for acute heart failure: A meta-analysis. Eur. J. Heart Fail..

[60] Rodríguez-Mañero M (2018). Validating left atrial low voltage areas during atrial fibrillation and atrial flutter using multielectrode automated electroanatomic mapping. JACC Clin. Electrophysiol..

[61] Gould SE, Day M, Jones SS, Doral H (2002). BMP-7 regulates chemokine, cytokine, and hemodynamic gene expression in proximal tubule cells. Kidney Int..

[62] Vordenbäumen S (2011). Altered serum levels of human neutrophil peptides (HNP) and human beta-defensin 2 (hBD2) in Wegener’s granulomatosis. Rheumatol. Int..

[63] Linscheid P, Seboek D, Zulewski H, Keller U, Müller B (2005). Autocrine/paracrine role of inflammation-mediated calcitonin gene-related peptide and adrenomedullin expression in human adipose tissue. Endocrinology.

[64] Aragón-Herrera A (2018). Relaxin activates AMPK-AKT signaling and increases glucose uptake by cultured cardiomyocytes. Endocrine.

[65] Takeda Y, Harada Y, Yoshikawa T, Dai P (2017). Direct conversion of human fibroblasts to brown adipocytes by small chemical compounds. Sci. Rep..

[66] Ouyang H, Luong P, Frödin M, Hansen SH (2020). p190A RhoGAP induces CDH1 expression and cooperates with E-cadherin to activate LATS kinases and suppress tumor cell growth. Oncogene.

[67] Liu J (2021). Cancer-associated fibroblasts provide a stromal niche for liver cancer organoids that confers trophic effects and therapy resistance. Cell. Mol. Gastroenterol. Hepatol..

[68] Yang JX (2019). Lipoxin A 4 ameliorates lipopolysaccharide-induced lung injury through stimulating epithelial proliferation, reducing epithelial cell apoptosis and inhibits epithelial-mesenchymal transition. Respir. Res..

[69] Salmani A (2019). A significant increase in expression of FOXP3 and IL-17 genes in patients with allergic rhinitis underwent accelerated rush immunotherapy. Iran. J. Basic Med. Sci..

[70] Paik WH (2014). Clobenpropit enhances anti-tumor effect of gemcitabine in pancreatic cancer. World J. Gastroenterol..

[71] Van Der Straaten HM (2004). Extra-domain-A fibronectin: A new marker of fibrosis in cutaneous graft-versus-host disease. J. Investig. Dermatol..

[72] Liu W (2020). MiR-200a regulates nasopharyngeal carcinoma cell migration and invasion by targeting MYH10. J. Cancer.

[73] Luo J (2017). Acetyl-CoA carboxylase rewires cancer metabolism to allow cancer cells to survive inhibition of the Warburg effect by cetuximab. Cancer Lett..

[74] Aragón-Herrera A (2021). Relaxin has beneficial effects on liver lipidome and metabolic enzymes. FASEB J..

