# Supplementary material for: Relaxin-2 plasma levels in atrial fibrillation are linked to inflammation and oxidative stress markers
Source: Sci Rep. 2022 Dec 24;12:22287. doi: 10.1038/s41598-022-26836-1 (PMC9789945; doi:10.1038/s41598-022-26836-1)
Supplement: Supplementary file 1 — Supplementary Information. [file 41598_2022_26836_MOESM1_ESM.docx]

**Supplementary Information**

**Supplementary Table S1**. Logistic regression. Dependent variable: Peripheral vein subgroups according to the median relaxin-2 distribution. **p*-value<0.05, ***p*-value<0.01, ****p*-value<0.001.

AHT: arterial hypertension; BMI: body mass index; Coeff.: coefficient; Gal-3: galectin-3; LA: left atrium; SE: standard error.

| Men | | | | | |
| --- | --- | --- | --- | --- | --- |
| Peripheral vein subgroups according to the median relaxin-2 distribution | | | | | |
|  |  | Coeff. |  | SE | *p*-value |
| (Intercept) |  | -0.287 |  | 2.698 | 0.915 |
| Age |  | 0.005 |  | 0.029 | 0.869 |
| BMI |  | 0.005 |  | 0.064 | 0.935 |
| AHT |  | -0.173 |  | 0.608 | 0.776 |
| LA Gal-3 |  | 0.021 |  | 0.038 | 0.573 |

**Supplementary Table S2**. Logistic regression. Dependent variable: Peripheral vein subgroups according to the median relaxin-2 distribution. **p*-value<0.05, ***p*-value<0.01, ****p*-value<0.001.

AHT: arterial hypertension; BMI: body mass index; Coeff.: coefficient; Gal-3: galectin-3; SE: standard error.

| Men | | | | | |
| --- | --- | --- | --- | --- | --- |
| Peripheral vein subgroups according to the median relaxin-2 distribution | | | | | |
|  |  | Coeff. |  | SE | *p*-value |
| (Intercept) |  | -0.558 |  | 2.744 | 0.839 |
| Age |  | 0.005 |  | 0.029 | 0.864 |
| BMI |  | -0.003 |  | 0.065 | 0.964 |
| AHT |  | -0.130 |  | 0.609 | 0.830 |
| Peripheral vein Gal-3 |  | 0.053 |  | 0.051 | 0.300 |

**Supplementary Table S3**. Logistic regression. Dependent variable: Left atrium subgroups according to the median relaxin-2 distribution. **p*-value<0.05, ***p*-value<0.01, ****p*-value<0.001.

AHT: arterial hypertension; BMI: body mass index; Coeff.: coefficient; DEFA3: alpha defensin 3; LA: left atrium; SE: standard error.

| Men | | | | | |
| --- | --- | --- | --- | --- | --- |
| Left atrium subgroups according to the median relaxin-2 distribution | | | | | |
|  |  | Coeff. |  | SE | *p*-value |
| (Intercept) |  | 12.910 |  | 5.929 | **0.029** |
| Age |  | 0.005 |  | 0.031 | 0.873 |
| BMI |  | -0.020 |  | 0.068 | 0.768 |
| AHT |  | -0.073 |  | 0.649 | 0.910 |
| LA DEFA3 |  | -6.686 |  | 2.483 | **0.007** |

**Supplementary Table S4**. Logistic regression. Dependent variable: Peripheral vein subgroups according to the median relaxin-2 distribution. **p*-value<0.05, ***p*-value<0.01, ****p*-value<0.001.

AHT: arterial hypertension; BMI: body mass index; Coeff.: coefficient; DEFA3: alpha defensin 3; LA: left atrium; SE: standard error.

| Men | | | | | |
| --- | --- | --- | --- | --- | --- |
| Peripheral vein subgroups according to the median relaxin-2 distribution | | | | | |
|  |  | Coeff. |  | SE | *p*-value |
| (Intercept) |  | 15.971 |  | 6.356 | **0.012** |
| Age |  | -0.009 |  | 0.032 | 0.773 |
| BMI |  | -0.023 |  | 0.071 | 0.748 |
| AHT |  | 0.352 |  | 0.670 | 0.599 |
| LA DEFA3 |  | -7.964 |  | 2.651 | **0.003** |

**Supplementary Table S5**. Logistic regression. Dependent variable: Left atrium subgroups according to the median relaxin-2 distribution. **p*-value<0.05, ***p*-value<0.01, ****p*-value<0.001.

AHT: arterial hypertension; BMI: body mass index; Coeff.: coefficient; IL-6: interleukin-6; LA: left atrium; SE: standard error.

| Men | | | | | | |
| --- | --- | --- | --- | --- | --- | --- |
| Left atrium subgroups according to the median relaxin-2 distribution | | | | | | |
|  |  | Coeff. |  | SE |  | *p*-value |
| (Intercept) |  | 9.365 |  | 5.889 |  | 0.112 |
| Age |  | 0.027 |  | 0.030 |  | 0.376 |
| BMI |  | 0.012 |  | 0.065 |  | 0.857 |
| AHT |  | -0.277 |  | 0.629 |  | 0.659 |
| LA IL-6 |  | -7.359 |  | 3.615 |  | **0.042** |

**Supplementary Table S6**. Logistic regression. Dependent variable: Peripheral vein subgroups according to the median relaxin-2 distribution. **p*-value<0.05, ***p*-value<0.01, ****p*-value<0.001.

AHT: arterial hypertension; BMI: body mass index; Coeff.: coefficient; IL-6: interleukin-6; LA: left atrium; SE: standard error.

| Men | | | | | | |
| --- | --- | --- | --- | --- | --- | --- |
| Peripheral vein subgroups according to the median relaxin-2 distribution | | | | | | |
|  |  | Coeff. |  | SE |  | *p*-value |
| (Intercept) |  | 12.508 |  | 6.374 |  | 0.050 |
| Age |  | 0.017 |  | 0.030 |  | 0.580 |
| BMI |  | 0.015 |  | 0.066 |  | 0.827 |
| AHT |  | 0.123 |  | 0.641 |  | 0.848 |
| LA IL-6 |  | -9.295 |  | 3.929 |  | **0.018** |

**Supplementary Table S7**. Logistic regression. Dependent variable: Peripheral vein subgroups according to the median relaxin-2 distribution. **p*-value<0.05, ***p*-value<0.01, ****p*-value<0.001.

AHT: arterial hypertension; BMI: body mass index; Coeff.: coefficient; SE: standard error.

| Men | | | | | | | |
| --- | --- | --- | --- | --- | --- | --- | --- |
| Peripheral vein subgroups according to the median relaxin-2 distribution | | | | | | | |
|  |  | Coeff. |  | SE |  | *p*-value | |
| (Intercept) |  | 1.189 |  | 2.888 |  | | 0.680 |
| Age |  | -0.003 |  | 0.030 |  | | 0.910 |
| BMI |  | -0.002 |  | 0.066 |  | | 0.975 |
| AHT |  | 0.228 |  | 0.645 |  | | 0.723 |
| Peripheral vein H_2_O_2_ |  | -6.175 |  | 3.064 |  | | **0.044** |

**Supplementary Table S8**. Spearman correlation between left atrium and peripheral vein relaxin-2 plasma levels with different biomarkers in all AF patients studied. The exact *n* values are stated in the table depending of the condition studied. Statistical analysis: Spearman bivariate correlation analysis. **p*-value<0.05, ***p*-value<0.01, ****p*-value<0.001. AF: atrial fibrillation; DEFA3: alpha defensin 3; Gal-3: galectin-3; IL-6: interleukin-6; LA: left atrium.

| **AF patients (n=68)** | | | |
| --- | --- | --- | --- |
|  |  | Left atrium relaxin-2 plasma levels (pg/mL) | Peripheral vein relaxin-2 plasma levels (pg/mL) |
| LA Gal-3 (ng/mL) | Correlation coefficient | 0.245 | 0.251 |
|  | *p*-value | 0.051 | **0.045** |
|  | n | 64 | 64 |
| Peripheral vein Gal-3 (ng/mL) | Correlation coefficient | 0.276 | 0.282 |
|  | *p*-value | **0.027** | **0.024** |
|  | n | 64 | 64 |
| LA DEFA3 (a.u.) | Correlation coefficient | -0.353 | -0.426 |
|  | *p*-value | 0.003 | **0.000** |
|  | n | 68 | 68 |
| LA IL-6 (a.u.) | Correlation coefficient | -0.412 | -0.567 |
|  | *p*-value | **0.001** | **0.000** |
|  | n | 67 | 67 |
| LA H_2_O_2_ (µM) | Correlation coefficient | -0.206 | -0.286 |
|  | *p*-value | 0.102 | **0.022** |
|  | n | 64 | 64 |
| Peripheral vein H_2_O_2_ (µM) | Correlation coefficient | -0.159 | -0.311 |
|  | *p*-value | 0.208 | **0.012** |
|  | n | 64 | 64 |

**Supplementary Table S9**. Spearman correlation between left atrium and peripheral vein relaxin-2 plasma levels with different biomarkers in AF patients with sinus rhythm or AF rhythm. The exact *n* values are stated in the table depending of the condition studied. Statistical analysis: Spearman bivariate correlation analysis. **p*-value<0.05, ***p*-value<0.01, ****p*-value<0.001. AF: atrial fibrillation; DEFA3: alpha defensin 3; Gal-3: galectin-3; IL-6: interleukin-6; LA: left atrium.

| **AF patients with sinus rhythm (n=45)** | | | |
| --- | --- | --- | --- |
|  |  | Left atrium relaxin-2 plasma levels (pg/mL) | Peripheral vein relaxin-2 plasma levels (pg/mL) |
| LA Gal-3 (ng/mL) | Correlation coefficient | 0.282 | 0.412 |
|  | *p*-value | 0.066 | **0.006** |
|  | n | 43 | 43 |
| Peripheral vein Gal-3 (ng/mL) | Correlation coefficient | 0.238 | 0.368 |
|  | *p*-value | 0.125 | **0.015** |
|  | n | 43 | 43 |
| LA DEFA3 (a.u.) | Correlation coefficient | -0.267 | -0.449 |
|  | *p*-value | 0.076 | **0.002** |
|  | n | 45 | 45 |
| LA IL-6 (a.u.) | Correlation coefficient | -0.374 | -0.532 |
|  | *p*-value | **0.012** | **0.000** |
|  | n | 44 | 44 |
| LA H_2_O_2_ (µM) | Correlation coefficient | -0.199 | -0.300 |
|  | *p*-value | 0.212 | 0.057 |
|  | n | 41 | 41 |
| Peripheral vein H_2_O_2_ (µM) | Correlation coefficient | -0.111 | -0.252 |
|  | *p*-value | 0.489 | 0.112 |
|  | n | 41 | 41 |
| **AF patients with AF rhythm (n=19)** | | | |
|  |  | Left atrium relaxin-2 plasma levels (pg/mL) | Peripheral vein relaxin-2 plasma levels (pg/mL) |
| LA Gal-3 (ng/mL) | Correlation coefficient | 0.212 | -0.098 |
|  | *p*-value | 0.414 | 0.708 |
|  | n | 17 | 17 |
| Peripheral vein Gal-3 (ng/mL) | Correlation coefficient | 0.134 | 0.005 |
|  | *p*-value | 0.609 | 0.985 |
|  | n | 17 | 17 |
| LA DEFA3 (a.u.) | Correlation coefficient | -0.248 | -0.108 |
|  | *p*-value | 0.305 | 0.659 |
|  | n | 19 | 19 |
| LA IL-6 (a.u.) | Correlation coefficient | 0.313 | -0.112 |
|  | *p*-value | 0.192 | 0.646 |
|  | n | 19 | 19 |
| LA H_2_O_2_ (µM) | Correlation coefficient | -0.166 | -0.202 |
|  | *p*-value | 0.497 | 0.407 |
|  | n | 19 | 19 |
| Peripheral vein H_2_O_2_ (µM) | Correlation coefficient | 0.119 | -0.307 |
|  | *p*-value | 0.628 | 0.200 |
|  | n | 19 | 19 |


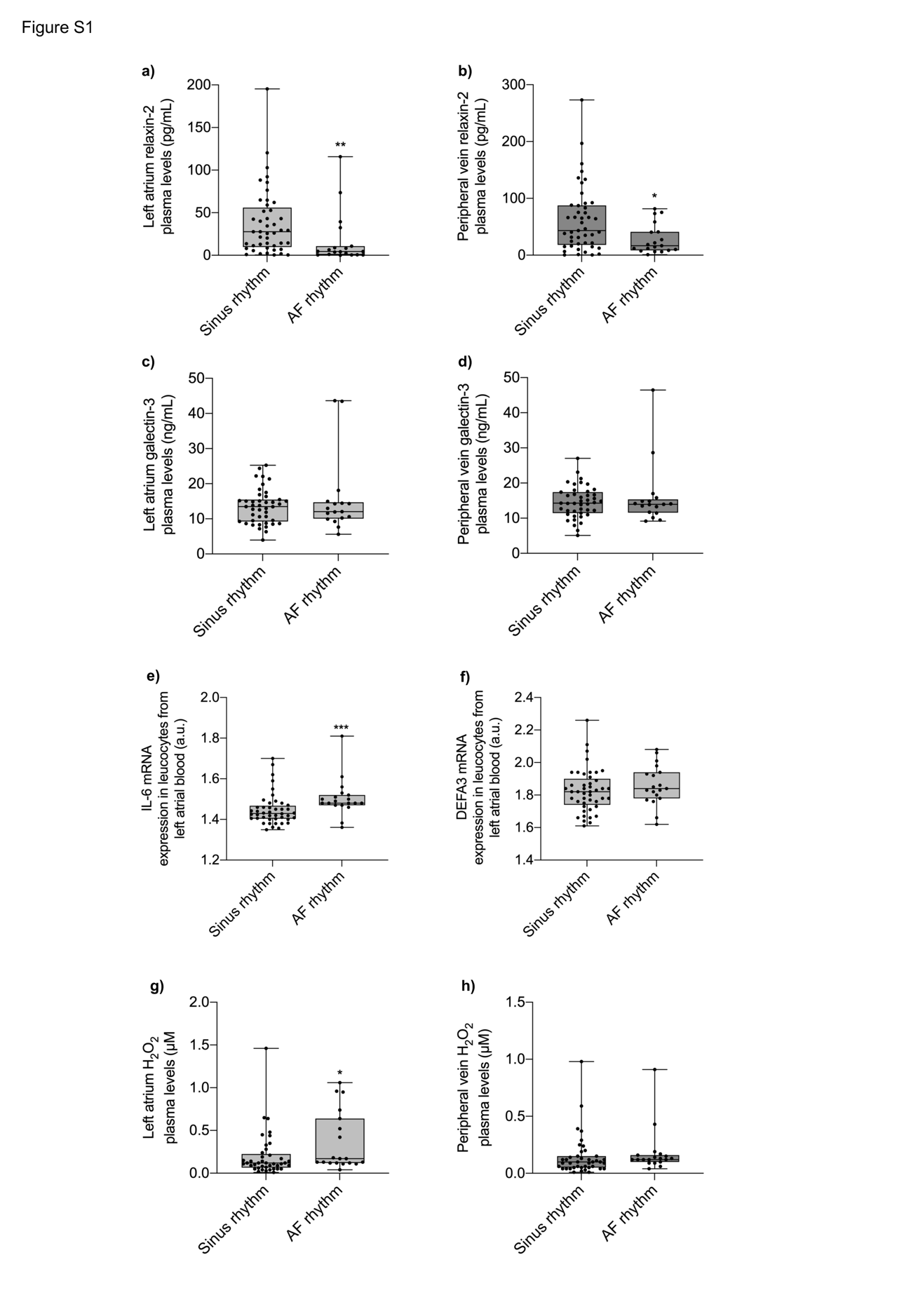


**Supplementary Figure S1**. Boxplot showing left atrium and peripheral vein relaxin-2, galectin-3, and H_2_O_2_ plasma levels, and IL-6 and DEFA3 mRNA expression in leucocytes from left atrial plasma regarding sinus or AF rhythm in AF patients. Left atrium and peripheral vein relaxin-2 plasma levels are significantly increased in AF patients with sinus rhythm compared with AF rhythm (n_sinus_ _rhythm_ = 45, n_AF rhythm_ = 19). IL-6 mRNA expression in leucocytes from left atrial plasma (n_sinus_ _rhythm_ = 44, n_AF rhythm_ = 19) and left atrial H_2_O_2_ plasma levels (n_sinus_ _rhythm_ = 41, n_AF rhythm_ = 19) are significantly increased in AF patients with AF rhythm compared with sinus rhythm Statistical analysis: Mann-Whitney U test. **p*-value<0.05, ***p*-value<0.01. AF: atrial fibrillation; DEFA3: alpha defensin 3; IL-6: interleukin-6.


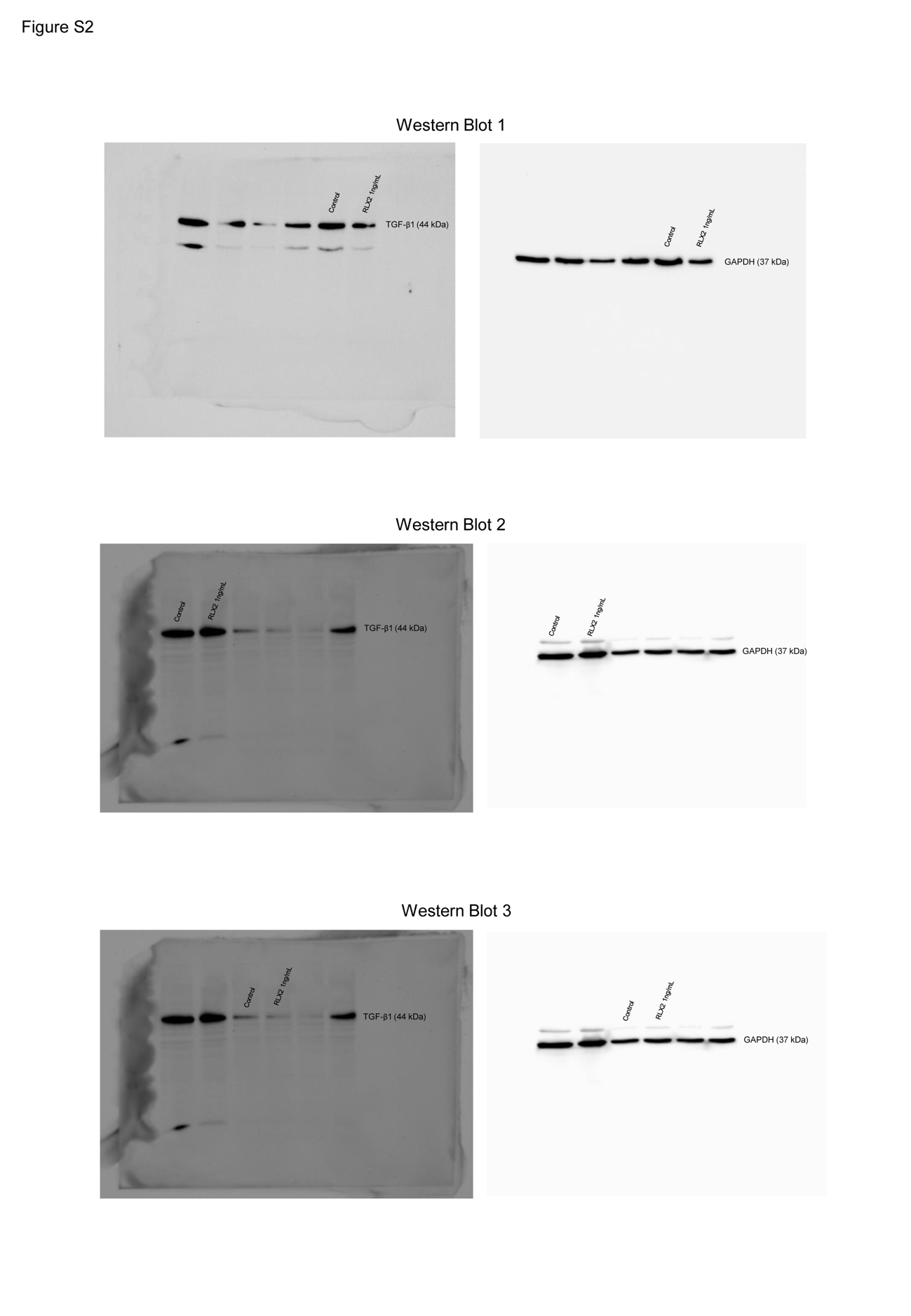


**Supplementary Figure S2**. Full blots of the pro-fibrotic molecule transforming growth factor-β1 (TGF-β1) protein expression in NHCF-A after recombinant human luteal relaxin-2 (RLX2) treatment for 24 hours of the western blot 1, 2 and 3 (n=3 replicates). Protein levels were normalized using glyceraldehyde-3-phosphate dehydrogenase (GAPDH).


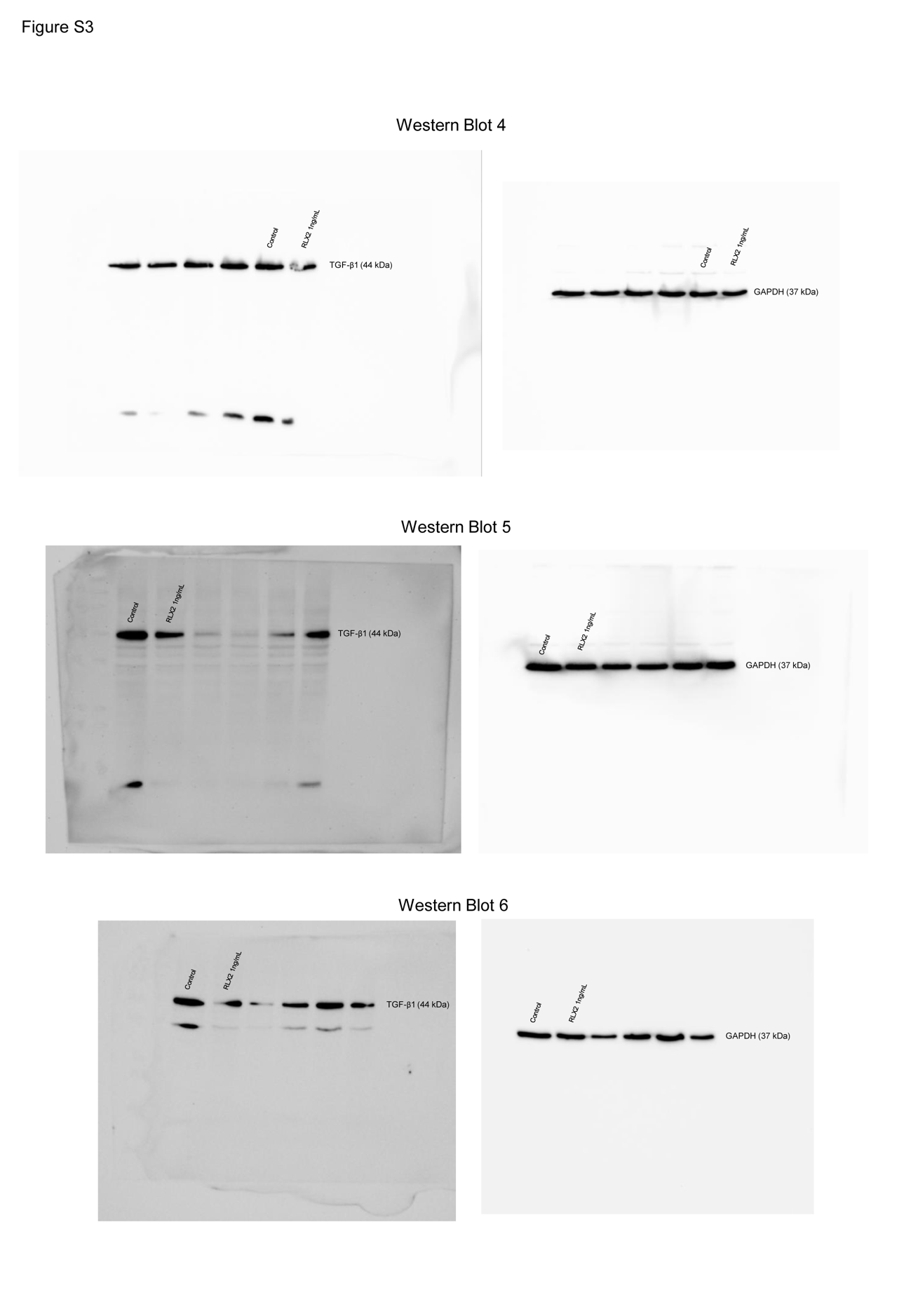


**Supplementary Figure S3**. Full blots of the pro-fibrotic molecule transforming growth factor-β1 (TGF-β1) protein expression in NHCF-A after recombinant human luteal relaxin-2 (RLX2) treatment for 24 hours of the western blot 4, 5 and 6 (n=3 replicates). Protein levels were normalized using glyceraldehyde-3-phosphate dehydrogenase (GAPDH).
